# Improved genome editing by an engineered CRISPR-Cas12a

**DOI:** 10.1093/nar/gkac1192

**Published:** 2022-12-20

**Authors:** Enbo Ma, Kai Chen, Honglue Shi, Elizabeth C Stahl, Ben Adler, Marena Trinidad, Junjie Liu, Kaihong Zhou, Jinjuan Ye, Jennifer A Doudna

**Affiliations:** Innovative Genomics Institute, University of California, Berkeley, CA 94720, USA; Department of Molecular and Cell Biology, University of California, Berkeley, CA 94720, USA; Innovative Genomics Institute, University of California, Berkeley, CA 94720, USA; Department of Molecular and Cell Biology, University of California, Berkeley, CA 94720, USA; Innovative Genomics Institute, University of California, Berkeley, CA 94720, USA; California Institute for Quantitative Biosciences (QB3), University of California, Berkeley, CA 94720, USA; Innovative Genomics Institute, University of California, Berkeley, CA 94720, USA; California Institute for Quantitative Biosciences (QB3), University of California, Berkeley, CA 94720, USA; Innovative Genomics Institute, University of California, Berkeley, CA 94720, USA; Department of Molecular and Cell Biology, University of California, Berkeley, CA 94720, USA; Innovative Genomics Institute, University of California, Berkeley, CA 94720, USA; Department of Molecular and Cell Biology, University of California, Berkeley, CA 94720, USA; Innovative Genomics Institute, University of California, Berkeley, CA 94720, USA; Department of Molecular and Cell Biology, University of California, Berkeley, CA 94720, USA; Howard Hughes Medical Institute, University of California, Berkeley, CA 94720, USA; Howard Hughes Medical Institute, University of California, Berkeley, CA 94720, USA; Innovative Genomics Institute, University of California, Berkeley, CA 94720, USA; Department of Molecular and Cell Biology, University of California, Berkeley, CA 94720, USA; California Institute for Quantitative Biosciences (QB3), University of California, Berkeley, CA 94720, USA; Howard Hughes Medical Institute, University of California, Berkeley, CA 94720, USA; Department of Chemistry, University of California, Berkeley, CA, USA; MBIB Division, Lawrence Berkeley National Laboratory, Berkeley, CA 94720, USA; Gladstone Institutes, University of California, San Francisco, CA 94114, USA

## Abstract

CRISPR-Cas12a is an RNA-guided, programmable genome editing enzyme found within bacterial adaptive immune pathways. Unlike CRISPR-Cas9, Cas12a uses only a single catalytic site to both cleave target double-stranded DNA (dsDNA) (*cis*-activity) and indiscriminately degrade single-stranded DNA (ssDNA) (*trans*-activity). To investigate how the relative potency of *cis*- versus *trans*-DNase activity affects Cas12a-mediated genome editing, we first used structure-guided engineering to generate variants of *Lachnospiraceae bacterium* Cas12a that selectively disrupt *trans*-activity. The resulting engineered mutant with the biggest differential between *cis*- and *trans*-DNase activity *in vitro* showed minimal genome editing activity in human cells, motivating a second set of experiments using directed evolution to generate additional mutants with robust genome editing activity. Notably, these engineered and evolved mutants had enhanced ability to induce homology-directed repair (HDR) editing by 2–18-fold compared to wild-type Cas12a when using HDR donors containing mismatches with crRNA at the PAM-distal region. Finally, a site-specific reversion mutation produced improved Cas12a (iCas12a) variants with superior genome editing efficiency at genomic sites that are difficult to edit using wild-type Cas12a. This strategy establishes a pipeline for creating improved genome editing tools by combining structural insights with randomization and selection. The available structures of other CRISPR-Cas enzymes will enable this strategy to be applied to improve the efficacy of other genome-editing proteins.

## INTRODUCTION

**Figure 1.**
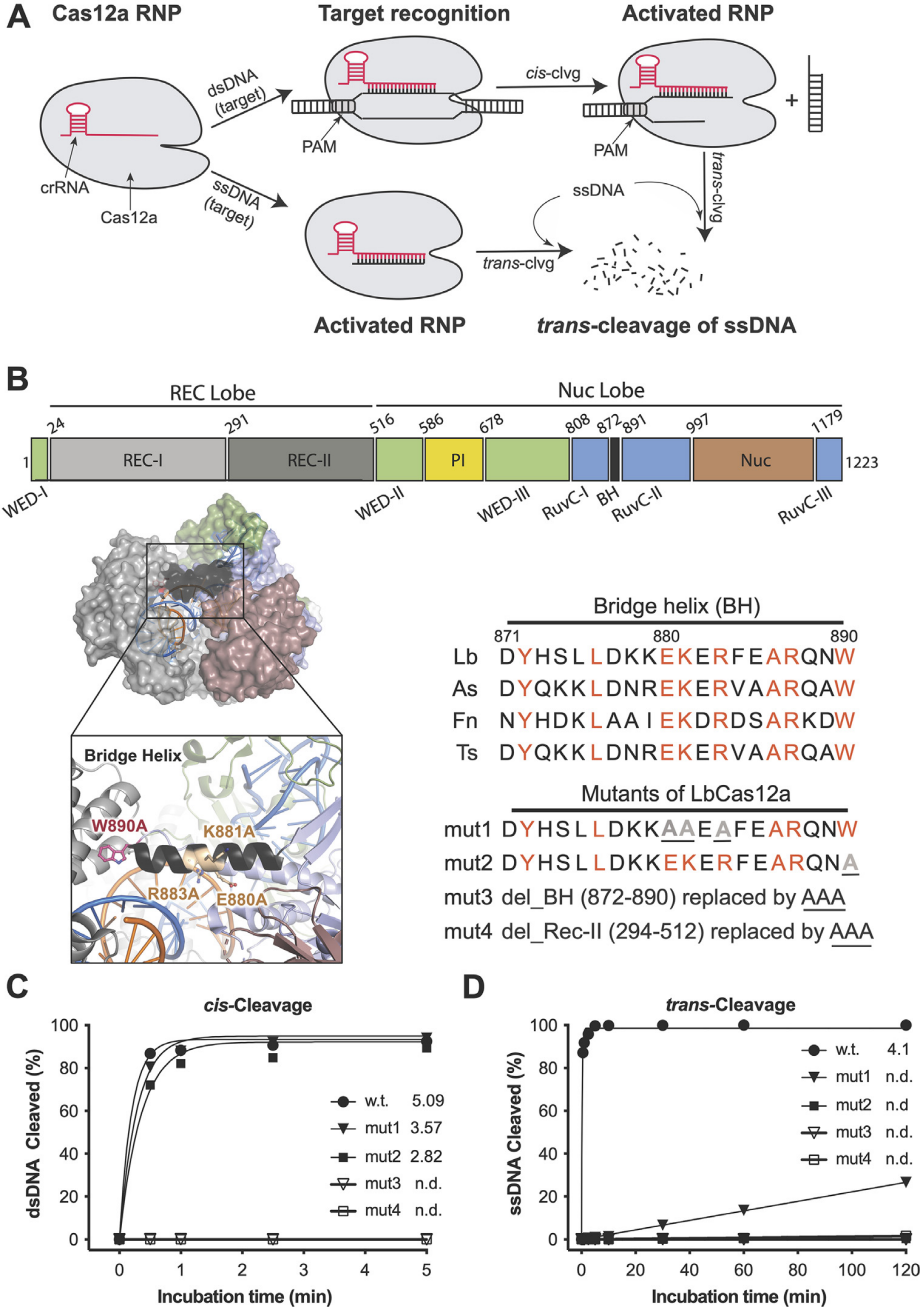
Importance of the bridge helix (BH) of LbCas12a protein in regulating its nuclease activities. (**A**) Illustration of *cis*- and *trans*-cleavage activities of Cas12a proteins. *Trans*-activity of Cas12a RNP can be activated by either direct binding to target ssDNA or after processing its target dsDNA. (**B**) Schematic presentation of LbCas12a protein. Domain assignment for LbCas12a (upper panel), protein structure (PDB ID: 5XUS) highlighting the bridge helix in LbCas12a (lower panel, left), and bridge helix sequences of different Cas12a orthologs and designed mutations from LbCas12a (lower panel, right). Point mutations are underlined, and deletions are replaced with a triple alanine sequence (AAA) which is also underlined. (**C**) and (**D**). *In vitro* kinetic studies of *cis*- and *trans*-cleavage activities by the wild-type (WT) protein and four designed mutants, mut1-4. Each data point is averaged from two independent assays. In the *cis*-cleavage assays, the non-target strand (NTS) of the dsDNA substrate was 5’-end-labeled with γ-^32^P-ATP. In the *trans*-cleavage assays, a ssDNA substrate was 5’-end-labeled with γ-^32^P-ATP. Initial reaction rates are given after each protein symbol; n.d., not detected.

CRISPR (clustered regularly interspaced short palindromic repeats)-Cas (CRISPR-associated proteins) systems were discovered as adaptive immune systems in prokaryotes, and several have been repurposed for programmable genome editing tools (1–4). Both Cas9 and Cas12a proteins have been widely deployed for programmable genome editing, each with distinct properties that could impact editing outcomes. A potentially important difference in the biochemical behavior between Cas9 and Cas12a is the ability of Cas12a to carry out dual nuclease activities: cleaving target double-stranded DNA (dsDNA) guided by CRISPR RNA (crRNA) (*cis*-activity) and non-specifically degrading single-stranded DNA (ssDNA) following crRNA-guided target DNA binding and cleavage (*trans*-activity) (Figure 1A). This *trans*-ssDNase activity has been harnessed for Cas12a-mediated nucleic acid detection and viral diagnosis (5–8). However, how and whether the *trans*-nuclease activity could impact genome editing outcomes are not yet fully understood.

Cas12a comprises three structural regions: the recognition (Rec) lobe, the nuclease (Nuc) lobe (including the RuvC, bridge helix (BH), and Nuc domains), and a connection region (including the Wedge (WED), and PAM-interacting (PI) domains) between the two lobes (9–11) (Figure 1A). It has been shown that Cas12a uses a single active site located in the RuvC domain to cleave both strands of the target dsDNA in *cis-*activity as well as non-specific ssDNA in *trans-*activity, following a well-defined order in the reaction steps (11–14). When CRISPR-Cas12a binds the target DNA, Cas12a undergoes a closed-to-open conformational change (13) and the spacer region of the crRNA forms Watson-Crick base pairs with its complementary target strand (TS) in the target DNA to form an R-loop. Upon R-loop formation, the non-target strand (NTS) is immediately accessed by the active site in the RuvC domain, nicked, and trimmed by five nucleotides, which enables the TS to reach the active site and to be cleaved (13,14). After cleavage, the TS is released from the active site to enable *trans*-cleavage of non-specific ssDNA. It has been shown that *trans* ssDNA cleavage requires NTS cleavage and can be accelerated by both NTS trimming and TS cleavage (13,14). Given this sequential pathway of Cas12a reaction steps, it seemed possible that Cas12a's *trans*-cleavage activity might be inhibited or reduced with minimal effect on target-specific *cis*-cleavage activity.

To investigate the effect of disruptions in Cas12a's dual nuclease activity on genome editing, we employed a combination of structure-guided engineering and directed evolution to identify Cas12a variants that minimize *trans*-DNase activity while retaining robust *cis*-dsDNA cutting behavior. In principle, Cas12a proteins with reduced *trans*-activity might improve DNA integration efficiency as an editing outcome following homology-directed repair (HDR) since *trans*-activity could potentially degrade ssDNA donor templates used for HDR.

A previous biochemical and single-molecule study showed that perturbations at the bridge helix, which resides between the RuvC and Rec-II domains and is responsible for RuvC activation, minimally impacted Cas12a's *cis*-cleavage activity but reduced the NTS trimming activity in *Francisella novicida* Cas12a (FnCas12a) (15). We therefore hypothesized that mutations in the BH region might disfavor *trans*-cutting while maintaining target-specific *cis-*cleavage activity. Using structural-based engineering, we found that a W890A mutation in the BH of *Lachnospiraceae bacterium ND2006* Cas12a (LbCas12a) abolished *trans*-activity while modestly reducing *cis*-activity. Combined with beneficial mutations identified by directed evolution, we produced Cas12a variants with both high-level *cis*-nuclease activity and minimized *trans*-ssDNase activity. Compared to wild-type Cas12a, these new variants showed elevated HDR efficiency when using DNA donors containing mismatches with crRNA at the PAM-distal region. Surprisingly, the restoration of W890 in these beneficial variants created improved Cas12a (iCas12a) proteins that possess significantly enhanced *cis*- and *trans*-activity *in vitro* and improved genome editing efficiency in human cells relative to the original Cas12a protein. This study demonstrates that the dual activities of CRISPR-Cas12a can be altered to enhance its application for both genome manipulation and nucleic acid detection.

## MATERIALS AND METHODS

### Preparation of designed CRISPR-cas12a mutants

The mutants of CRISPR *Lachnospiraceae bacterium* Cas12a (LbCas12a) bearing mutations in the bridge helix (BH) domain were generated by site-directed mutagenesis polymerase chain reaction (PCR) in the presence of a given wild-type LbCasd12a plasmid, two corresponding primers (synthesized by IDT) and KOD polymerase (Sigma-Aldrich). Specifically, the reaction was carried out in a 25 μl reaction containing 10 ng of wild-type LbCas12a plasmid and 0.75 μl of 10 μM primers containing desired mutations. After PCR, the reaction was treated with 1μl of DpnI (NEB, New England BioLabs) for 1 hour at 37°C before transformation. The wild-type LbCas12a plasmid is a home-made pET-based expression vector containing an N-terminal His_10_-tag, maltose-binding protein (MBP), and TEV protease cleavage site (QB3 MacroLab, UC Berkeley). The sequences of all the plasmid constructs are confirmed via Sanger sequencing (UC Berkeley DNA Sequencing Facility).

### Generation of new LbCas12a mutants via directed evolution

To engineer the mutant LbCas12a, mut2, for higher nuclease activities, we performed directed evolution using a bacteria selection system (Figure 3A). First, we made a chloramphenicol-resistant (CAM^+^) bacterial expression construct containing both point mutation of W890A (LbCas12a-mut2) and a guide crRNA that targets its complementary sequence containing protospacer adjacent motif (PAM) of TTTG in the *ccdB* toxin gene of the selection plasmid. This inducible *ccdB* gene is constructed in a plasmid with an ampicillin-resistant (Amp^+^) gene, and its expression is controlled by the arabinose-promoter pBAD for a positive selection. To simplify the engineering, we divided the LbCas12-mut2 protein into three regions (as shown in Figure 3B): region 1 (R1: 1–532), region 2 (R2: 507–820), and region 3 (R3: 797–1228). To generate mutant libraries of each region, we first performed error-prone PCR of the target region in the presence of the mut2 LbCas12a plasmid and controlled an error rate at 6- to 9-nucleotide mutations per kilobase by adding 0.24 mM MnCl_2_ in the PCR reaction. Specifically, the error-prone PCR was carried out with the ThermoTaq DNA polymerase (M0267S, NEB) in a reaction of 100 μl with 10 μl of 10X ThermoPol reaction buffer, 2 μl of 10 mM primers, 2.4 μl of 10 mM MnCl_2_, 32 ng of mut2 LbCas12a plasmid and 1 μl of ThermoTaq DNA Polymerase. At the same time, we used KOD polymerase to amplify the corresponding backbone, which is minus the target region, from the founder plasmid of mut2 LbCas12a. After purification of the error-prone target region and its corresponding backbone in agarose gel, the two purified products are ligated via Gibson Mix (NEB, New England BioLab) to generate an error-prone library, and the ligated product was further purified in agarose gel before electroporation.

Two nanograms of the error-prone library plasmid DNA were electroporated in 50 μl of competent cells made from *Escherichia coli* strain *BW25141(λDE3)* that contains the selection plasmid encoding the arabinose-inducible *ccdB* toxin gene. After recovery of the electroporated bacteria in 2 ml of SOB for 50 min at 37°C, 5 μl of the bacteria culture was plated onto a Petri agar-dish containing only CAM (as control), and the remainder culture was concentrated and plated on another Petri agar-dish containing both arabinose and CAM. Positive colonies that grew on the plate containing both arabinose and CAM were collected and replated. Plasmids of individual colonies from the replated plate were then prepared and sequenced. In this study, two rounds of selection were carried out.

### Protein expression and purification

The protocol for the purification of LbCas12a proteins has been described elsewhere (5). Simply, all the Cas12a proteins were expressed in *E. coli* Rosetta (DE3) cells (Sigma-Aldrich) cultured in Terrific Broth (TB) medium (Thermo Fisher Scientific) supplemented with the antibiotics of ampicillin and chloramphenicol. The cultivation was carried out at 37°C after inoculation with overnight-cultured starter at a 1:40 ratio. When the optical density (OD_600_) of the culture reached 0.6–0.8, the expression of LbCas12a was induced by the addition of isopropyl β-d-1-thioglalacctopyranoside (IPTG) to a final concentration of 0.1 mM and incubated overnight at 16°C. To purify the Cas12a proteins, the cultured cells were harvested and resuspended in Lysis Buffer (LB: 50 mM Tris–HCl, pH 7.5, 500 mM NaCl, 5% (v/v) glycerol, 1 mM TCEP, 0.5 mM PMSF and 0.25 mg/ml lysozyme as well as a cOmplete Protease Inhibitor Cocktail Tablet (Millipore Sigma) for every 50 ml), disrupted by sonication and centrifuged for 60 min at 18 000 rcf. The supernatant was incubated with Ni-NTA resin for 60 min at 4°C to pull down the His-tagged LbCas12a protein. After cleavage with TEV protease (lab synthesized) overnight at 4°C, the LbCas12a proteins were separated from His-tagged MBP via HiTrap Ni-NTA column (GE) and further purified over a HiTrap Heparin HP column (GE). The final gel filtration step (Superdex 200, GE) was carried out in gel-filtration buffer containing 20 mM Tris–HCl, pH 7.5, 200 mM NaCl, 5% (v/v) glycerol, and 1 mM TCEP.

### Nucleic acid preparation

All the DNA and RNA oligos used in this study were purchased from Integrated DNA Technologies, Inc. (IDT) and HPLC or PAGE-purified. For genome editing, the crRNAs and donor DNAs were synthesized from IDT DNA Technologies with chemical modifications provided by IDT to improve their stability and editing efficiency in cells. All the DNAs and crRNA sequences are listed in Supplementary Table 1.

For *in vitro* cleavage assays, the following guide crRNA and Oligo DNAs were listed below:

crRNA: 5′-UAAUUUCUACUAAGUGUAGAUGAUCGUUACGCUAACUAUGAGGGC

Non-target strand (NTS):

5′-GACGACAAAACTTTAGATCGTTACGCTAACTATGAGGGCTGTCTGTGGAATGCTA

Target strand (TS):

5′-TAGCATTCCACAGACAGCCCTCATAGTTAGCGTAACGATCTAAAGTTTTGTCGTC

DNA oligo used for *in vitro trans*-cleavage assays:

5′-CGCACGTACTGTAATCGCCAAACAGAGTAGAAATACGCAGAGCATGAGCG

### DNA cleavage assays

In DNA cleavage assays, the labeled strand was either fluorescently labeled at 5’-end with fluorophores of 5’6-FAM or radiolabeled at 5’-end with γ-^32^P-ATP, as stated in figure legends. For the formation of a dsDNA substrate, the 5′-end-labeled target or non-target strand was annealed with excess corresponding strand (a molar ratio of 1:1.2).

A typical *cis*-cleavage assay (unless otherwise stated) was carried out with 30 nM protein, 36 nM of crRNA, and 10 nM of 5’-FAM-labeled target DNA (or 4 nM of γ-^32^P-ATP 5’-end labeled target) in the cleavage buffer consisting of 20 mM HEPES (pH 7.5), 150 mM KCl, 10 mM MgCl_2_, 1% glycerol and 0.5 mM TCEP. Specifically, the protein and a guide crRNA were first incubated for 15 min at room temperature to form RNPs. Labeled DNA was added to the RNP, and the reaction was continuously carried out at 37°C for the durations indicated in the figures. For *trans*-cleavage assays, a typical reaction condition (unless otherwise stated) was the same as *cis*-cleavage reactions except that 45 nM unlabeled target dsDNA as an activator instead of labeled target dsDNA were used. After incubation for 30 min at 37°C, a labeled random ssDNA was added to the reactions, and the reaction was continuously incubated for the durations indicated in the figures. The reactions were quenched with DNA loading buffer (45% formamide and 15 mM EDTA, with a trace amount of xylene cyanol and bromophenol blue). After denatured at 95°C for 3 min, the cleavage products were separated in 15% urea–PAGE gel and quantified with Typhoon (Amersham, GE Healthcare).

For time course studies, the data were fitted to a curve generated by the formula of ${{Y\;}} = {{{Y}}_{{\rm{max}}}}{\rm{\;}} \times ( {1 - {{\rm{e}}^{ - {{kt}}}}} )$, where ${{\rm{Y}}_{{\rm{max}}}}$ is the pre-exponential factor, *k* is the rate constant (initial rate, min^−1^), and *t* is the reaction time (minutes).

### Cell culture and genomic editing

To evaluate the genome editing activity of different LbCas12a mutant proteins, we first assembled their RNPs with corresponding crRNAs before performing nucleofection with HEK293T cells or tdTomato neural progenitor cells (NPCs). Specifically, the protein (100 pmol) and the crRNA (120 pmol) with a molar ratio of 1:1.2 were first incubated for 15–25 min at room temperature to form RNPs and then mixed with a ssDNA electroporation enhancer (80pmol, purchased from IDT) with a molar ratio of 1:0.8 (RNP:enhancer) (the electroporation enhancer was used for all the cell editing experiments based on nucleofection unless otherwise noted). For HDR experiments, ssDNA donor template (100 pmol) was further added to the RNP solution with a molar ratio of 1:1 (RNP:donor ssDNA). Lonza SF (for HEK293T cells) and P3 (for tdTomato NPCs) buffers were used for the preparation of nucleofection mixtures.

HEK293T cells (UC Berkeley Cell Culture Facility) were cultured using Dulbecco's modification of Eagle's medium (DMEM) with l-glutamine, 4.5 g/l glucose, and sodium pyruvate (Corning) plus 10% FBS and penicillin and streptomycin (Gibco). Nucleofection of HEK293T cells with RNPs was performed using Lonza (Allendale, NJ) SF cell kits in an Amaxa 96-well Shuttle system with a program code CM-130. Each nucleofection reaction consisted of ∼2.0×10^5^ cells and 100 pmol RNP with a total volume of 25 μl in the supplemented SF buffer according to the Lonza protocol. After nucleofection, 75 μl of growth media was added to each nucleofection cuvette to transfer the cells to 12-well tissue culture plates with a total culture volume of 1ml/well. For HDR experiments, HDR enhancer (Alt-R™ HDR Enhancer V2, 0.69 mM stock in DMSO, purchased from IDT) with a final concentration of 0.33 μM was added to the culture media. After incubation at 37°C for 24 h, the cell culture media was refreshed. The HEK293T cells were harvested for analysis after further incubation at 37°C for 72 h.

NPCs were isolated from embryonic day 13.5 Ai9-tdTomato homozygous mouse brains (16). Cells were cultured as neurospheres in NPC medium: DMEM/F12 with glutamine, Na-Pyruvate, 10 mM HEPES, non-essential amino acid, penicillin and streptomycin (100×), 2-mercaptoethanol (1000×), B-27 without vitamin A, N2 supplement, and growth factors, bFGF and EGF (both 20 ng/ml as final concentration). NPCs were passaged using MACS Neural Dissociation Kit (Papain, CAT# 130-092-628, Miltenyi Biotec) following manufacturer's protocol. bFGF and EGF were refreshed every three days and cells were passaged every 6 days. The NPC line was authenticated by immunocytochemistry marker staining for Nestin and GFAP. Nucleofection of NPCs with RNP was performed using Lonza (Allendale, NJ) P3 cell kits in an Amaxa 96-well Shuttle system with a program code EH-100. Each nucleofection reaction consisted of ∼2.5×10^5^ cells and 100 pmol RNP with a total volume of 20 μl in the supplemented P3 buffer according to the Lonza protocol. After nucleofection, 80 μl of growth media was added to the nucleofection cuvette and 15 μl of NPC culture was then transferred to 96-well tissue culture plates pre-coated (using laminin, fibronectin, and poly-dl-ornithine), with a total culture volume of 100 μl/well. After incubation at 37°C for 72 h, the cell culture media was refreshed. The NPCs were harvested for analysis after further incubation at 37°C for 72 h.

For genomic DNA extraction, the media was removed by aspiration, and 100μl of Quick Extraction solution (Epicentre, Madison, WI) was added to lyse the cells (65°C for 20 min and then 95°C for 20 min). The concentration of genomic DNA was determined by NanoDrop, and the cell lysate was stored at −20°C. tdTomato-positive NPCs were analyzed by flow cytometry.

To fully investigate target editing (TE) and HDR of each LbCas12a protein on a genomic DNA target in HEK293T cells, target amplicons were PCR-amplified in the presence of corresponding primers which were designed to have no overlap with their corresponding donor ssDNA sequence in the case of HDR. The PCR products were purified with magnetic beads (Berkeley Sequencing Core Facility) before being subjected to next-generation sequencing (NGS) with MiSeq (Illumina) at 2 × 300 bp with a depth of at least 10 000 reads per sample (Innovative Genomics Institute Center for Translational Genomics).

The paired-end Illumina sequencing reads were trimmed using the BBDuk tool in Geneious Prime (https://www.geneious.com/prime) with a minimum quality of 20 and a minimum length of 20, and then merged using the BBmerge tool in Geneious Prime. The merged reads were then subjected to CRISPResso2 (https://github.com/pinellolab/CRISPResso2) to quantify the rate of indels and HDR with the two following commands, respectively:

CRISPResso –fastq_r1 MERGED_READS –amplicon_seq AMPLICON_SEQUENCE –guide_seq GUIDE_SEQUENCE -n nhej -wc -5 -w 9 –plot_window_size 20 -o OUTPUT_FILE

CRISPResso –fastq_r1 MERGED_READS –amplicon_seq AMPLICON_SEQUENCE –guide_seq GUIDE_SEQUENCE -e DONOR_SEQUENCE -wc -5 -w 9 –plot_window_size 20 -o OUTPUT_FILE, where *–fastq_r1* is followed by the path of the input merged fastq file, *–amplicon_seq* is followed by the full sequence of amplicon, *–guide_seq* is followed by the spacer sequence of the guide RNA, *–e* is followed by the expected amplicon sequence after successful HDR, *-wc* is followed by the quantification window center relative to the 3’ end of the spacer sequence, *-w* is followed by the size of the quantification window and *–plot_window_size* is followed by the size of the quantification window for visualizing the indels. The position and size of the quantification window were adjusted by the cleavage patterns of Cas12a mutants in this study.

### Protein thermostability assay

The reactions for analyzing the thermostability of LbCas12a proteins were performed based on the instruction from Protein Thermal Shift Dye kit (ThermoFisher Scientific). The protein melt fluorescent readings from CFX96 real-time System (BioRad) were directly recorded.

### Structural prediction

The full length of protein sequences of wild-type LbCas12a and its variants of mut2B-W, mut2C-W, and mut2C-WF were used for structure predictions with AlphaFold2 v.2.1.1 (17) using default parameters.

### Statistical analysis

For experiments performed with biological replicates, data are displayed as the average ± the standard error of the mean, with sample sizes provided in the figure legends. Statistical tests were performed using a one-way ANOVA and two-sided Dunnett's test to adjust *P*-values for multiple comparisons.

## RESULTS

### Cas12a bridge helix mutations differentially affect *cis*- versus *trans*-DNA cleavage

Data from mutagenesis and biochemical assays have shown that the disruption of the BH helical structure or the prevention of anchoring BH to the Rec-II domain in FnCas12a minimally reduces the *cis*-cleavage activity but impairs the NTS trimming activity (15). Furthermore, the NTS trimming for gap formation was shown to be a critical conformational prerequisite for the TS cleavage and for the subsequent *trans*-cleavage activity (13,14). Therefore, we rationalized that mutations in the BH might help us to dissect the *cis*- and *trans*-activities of Cas12a proteins. Sequence alignment of the bridge helix (BH) segments from several Cas12a orthologs revealed a high sequence identity (>40%) with several fully conserved residues (Figure 1B). For instance, in LbCas12a, we noticed that residues D877/E880 and R833/R887 in the BH build an acid-base network with K940 and E939 in the RuvC domain that may impact nuclease activity. Additionally, W890, which resides at the end of the BH and is anchored in a hydrophobic cavity of the Rec-II domain to stabilize the BH, could affect the closed-to-open conformational change of the whole protein (10,15,18).

We designed three sets of mutations in the BH of LbCas12a. Two of them are mutations at these highly conserved amino acid positions, named mut1 (E880A, K881A, and R883A) and mut2 (W890A), and another mutant with the entire BH replaced by a triple alanine sequence (AAA), named mut3 (ΔY872-W890). As the Rec-II domain is known to participate in the dynamic change and to directly interact with BH during the activity of Cas12a (11,18), an additional mutant, mut4 (ΔY294-T512), with the Rec-II domain replaced by AAA, was also included in our initial test (Figure 1B).

Proteins from the four designed mutants were purified (Supplementary Figure S1A) and were tested by *in vitro* cleavage assays using a 45nt CRISPR RNA (crRNA) containing a 21-base direct repeat region (loop domain) and a 24-base protospacer region complementary to the center of a 55-base pair (bp) dsDNA substrate in which the target region flanks the protospacer adjacent motif (PAM) of TTTA (Supplementary Figure S1B). We also tested target-activated ssDNA cleavage by these mutants and compared them to the wild-type enzyme. The biochemical assays showed that although bridge-helix mutants mut1 and mut2 retained relatively high levels of *cis*-cleavage activity on dsDNA substrate, their *trans*-ssDNase activity was substantially reduced or diminished (Figure 1C, D, Supplementary Figure S1C–F). However, mut3 and mut4, containing either entire BH deletion or REC-II domain truncation, showed no detectable *cis*- or *trans*-cleavage activity for either dsDNA or ssDNA substrates (Figure 1C, D, Supplementary Figure S1C–F), suggesting that the deletion of either the entire BH or Rec-II domain abolishes the enzyme functions.

**Figure 2.**
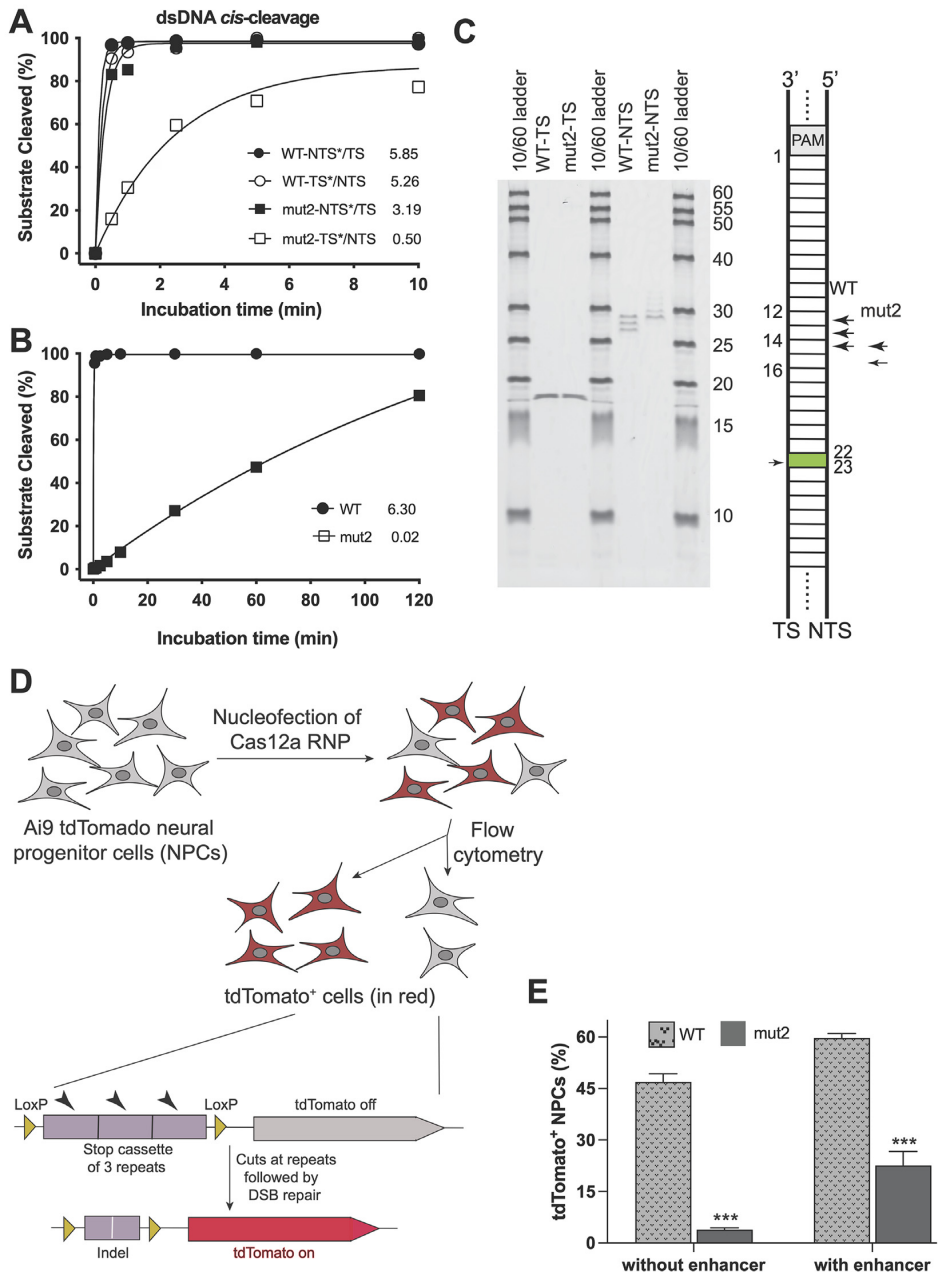
Mutational effect of W890A on the nuclease activities of LbCas12a. (**A**, **B**) Kinetic studies of the *cis*-cleavage activities on dsDNA (A) and ssDNA (B) by wild-type LbCas12a and mut2 (W890A). TS = target strand; NTS = nontarget strand; * = labeled strand. In these kinetic studies, the labeled strand was fluorescently labeled at the 5’-end with FAM. Each data point in panel (A) is averaged from three independent assays; each data point in panel (B) is averaged from two independent assays. Initial rates are presented after each symbol. (**C**) Mutational effect of W890A on the cleavage sites of NTS of dsDNA. (**D**) Genome editing workflow using tdTomato neural progenitor cells (NPCs) from Ai9 mice. The tdTomato gene will be turned on only when editing happens to remove the stop cassette. (**E**) Genome editing of NPCs by wild-type LbCas12a and mut2. The editing level is reflected by the percentage of tdTomato-positive NPCs (*n* = 3, means ± SD). In both delivery conditions, the editing efficiency of mut2 is significantly lower than the wild-type protein (***P < 0.001).

As Cas12a utilizes a single RuvC domain to cleave sequentially the two strands of dsDNA and then catalyze *trans*-ssDNA cutting (5,14,19,20), we tested how the dual activities of the RuvC domain are regulated with kinetic analyses of LbCas12a proteins with mutations in its bridge helix domain. From four variants that were tested (mut1-4), we chose mut2 LbCas12a (a point mutation of W890A) for further study due to its more substantially reduced *trans*-activity relative to mut1 (Figure 1D, Supplementary Figure S1E, F). Kinetic analysis of *cis*-cleavage was conducted using fluorescently 5’-end-labeled target (TS) or non-target strand (NTS) dsDNA substrates. Although the mutation of W890A in mut2 resulted in a ∼50% reduction in the initial rate (3.2 min^−1^ versus 5.9 min^−1^) of non-target strand (NTS) cleavage relative to the wild-type (WT) LbCas12a, the mut2-catalyzed TS cleavage rate was 10% that of the WT LbCas12a (0.5 min^−1^ versus 5.3 min^−1^) (Figure 2A, Supplementary Figure S2A, B). This mutational effect was even more evident with ssDNA substrates (Figure 2B, Supplementary Figure S2C, D). Analysis of the NTS and TS cleavage products showed that the mut2-generated NTS cleavage site was shifted by 2–3 nucleotides away from the PAM (Figure 2C) with reduced NTS trimming (Supplementary Figure S2A) relative to the wildtype protein NTS cleavage product, while the cleavage site on TS remained identical between these two proteins (Figure 2C). These results are consistent with previous biochemical studies of BH mutations in FnCas12a W971A, which is analogous to the LbCas12a W890A mutation (15,20). These findings support a ‘substrate-occlude model’, in which the altered NTS cleavage positions and reduced NTS trimming interferes with the subsequent entry of TS and non-specific ssDNA to the catalytic site, resulting in reduced TS *cis*-cleavage and diminished *trans*-activity (14).

The differentiated effect of W890A in mut2 on *cis*-cleavage and *trans*-cleavage in biochemical analyses made us wonder how this mutation would affect genome editing in mammalian cells. We tested this using neural progenitor cells (NPCs) isolated from Ai9 mice containing a tdTomato transgene under the control of a loxP-flanked stop cassette consisting of three repeating transcription terminators (16,21). LbCas12a-directed removal of the stop cassette would disrupt the terminator and activate the expression of tdTomato (Figure 2D). We compared genome editing induced by mut2 versus wild-type LbCas12a using a crRNA that targets the stop cassette and delivering these respective Cas12a ribonucleoproteins (RNPs) into NPCs by nucleofection. Flow cytometry data of tdTomato-positive cells harvested 6 days post nucleofection showed that mut2 was 3–10-fold less efficient at inducing genome editing relative to wild-type LbCas12a depending on the nucleofection protocol (Figure 2E). This result is consistent with the 10-fold reduction in the TS cleavage rate observed in our *in vitro* cleavage assays.

### Directed evolution of mutant LbCas12a to improve its genome editing activities

In general, Cas12a has been less widely used in mammalian genome editing compared to *Streptococcus pyogenes* Cas9 (SpyCas9) (22–24). Improvement of the enzymatic activity of LbCas12a could make this group of CRISPR proteins more advantageous for mammalian genome editing. Recent studies showed that the activity of Cas12a can be improved through protein engineering (25–27). In this study, we used directed evolution to select for beneficial mutants derived from mut2. The reduced DNA cleavage activities of mut2 over wild-type LbCas12 or mut1 was ideal as a starting point for gain-of-function selection.

**Figure 3.**
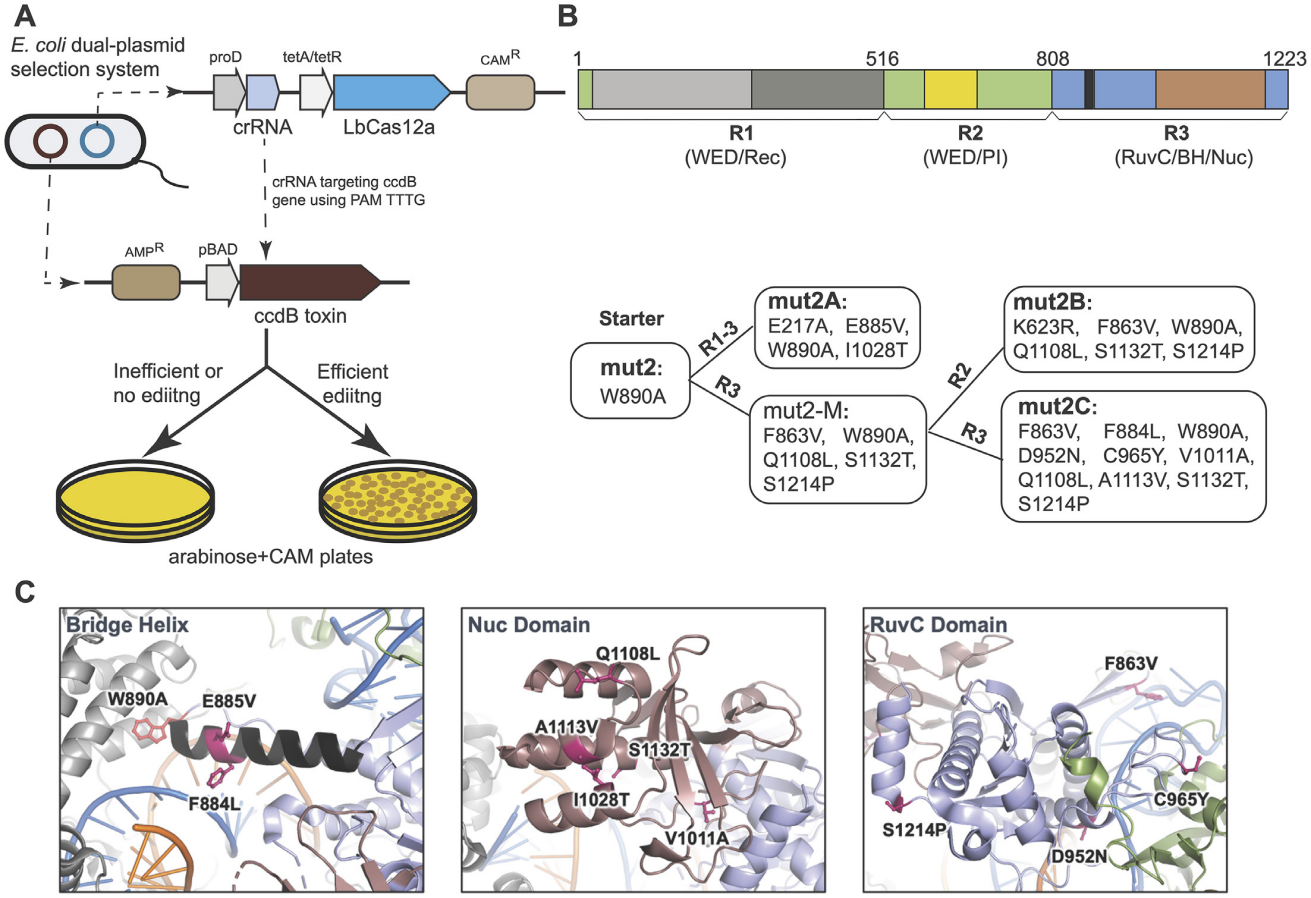
Enhancing the *cis*-cleavage activity of mut2 by directed evolution. (**A**) Schematic illustration of the positive selection system used for directed evolution of mut2 LbCas12a. (**B**) Generation of 3 beneficial mutants from mut2 (starter) by directed evolution. Upper panel showing the region (R) divisions of LbCas12a protein used for error-prone polymerase chain reaction (PCR) mutagenesis. Lower panel showing the mutants selected followed each round of mutagenesis. Three beneficial mutants (named mut2A, mut2B, and mut2C) were generated from two rounds of selection. The mutations in each variant are listed. (**C**) Snapshots of LbCas12a protein structure (PDB ID: 5XUS) highlighting the locations of the beneficial mutations obtained from directed evolution.

We employed a positive bacterial selection scheme, which was initially developed to evolve homing endonucleases (28) and later adapted for CRISPR-based genome editor engineering (25,29–32). In this approach, the positive selection of new Cas12a variants relies on Cas12a-mediated cleavage of a plasmid encoding the toxin-encoding *ccdB* gene under the control of an inducible promoter (Figure 3A). To reduce or minimize the background during selection, we selected a crRNA that targets the *ccdB* gene at the protospacer containing PAM of TTTG, which is a disfavored PAM *in vitro* by mut2-LbCas12a (Supplementary Figure S3A). When this selection scheme was tested with wild-type LbCas12a, nearly 100% survival of *E. coli* colonies was observed, whereas mut2 produced no bacterial survival (Supplementary Figure S3B). We then conducted directed evolution by randomly mutagenizing the entire mut2-encoding gene at a rate of 6- to 9-nucleotide mutations per kilobase. To simplify the screening process, we divided the entire protein sequence into three regions (R1, R2, and R3) for random mutagenesis carried out with error-prone polymerase chain reaction (PCR) (upper panel, Figure 3B). From two sequential rounds of evolution, three mutants (named mut2A, 2B, and 2C) showed nearly 100% bacterial survival under the selection system (Supplementary Figure S3C). Sanger sequencing showed that most of these beneficial mutations in the newly identified mutants are located in the Nuc lobe (including both RuvC and Nuc domains and BH) (lower panel, Figure 3B, C).

**Figure 4.**
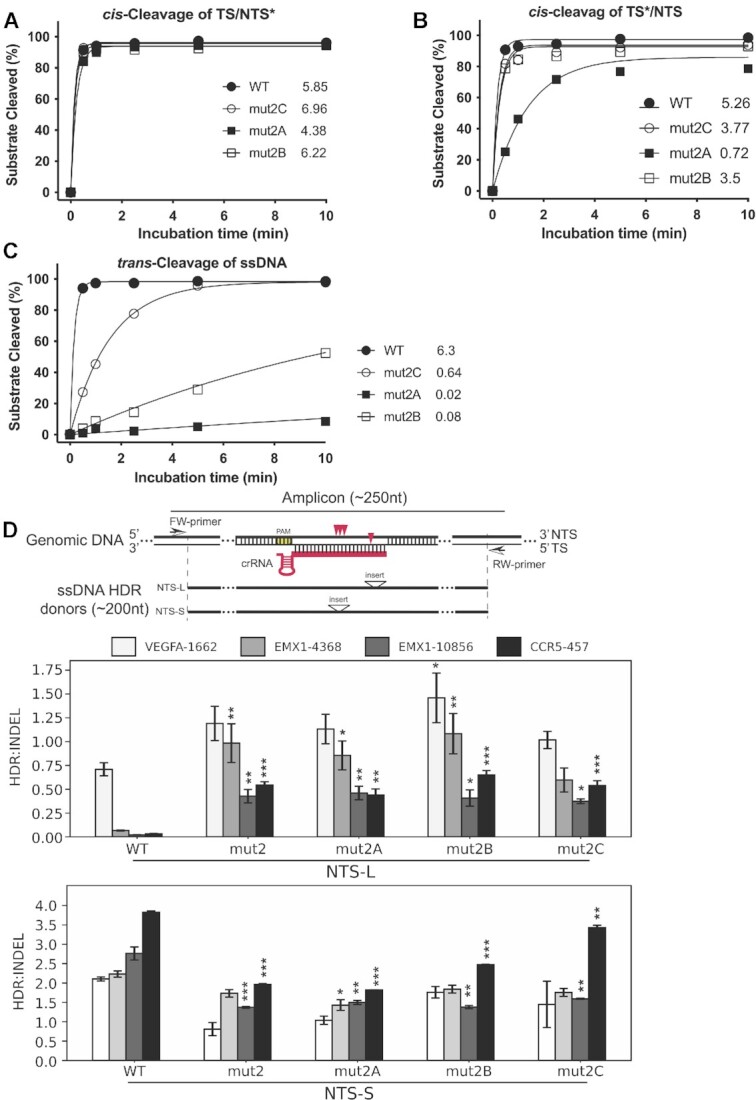
Enhanced activity of mut2A-C *in vitro* and *in cells*. (A–C) *In vitro* kinetic studies of the cleavage activities on NTS (**A**) and TS (**B**) in a dsDNA as well as the *trans*-cleavage activities (**C**). * indicates labeled strand. In these *in vitro* kinetic studies, the labeled strand was fluorescently labeled at the 5’-end with FAM. Each data point is averaged from three independent assays. All three beneficial mutants from directed evolution are more active than mut2. mut2B and mut2C display similar *cis*-activities on both strands as wild-type, but their *trans*-activities are dramatically lower relative to the wild-type. (**D**) Genome editing activity of mut2A-C in HEK293 cells. The upper panel shows the nontarget strand (NTS) ssDNA donors of NTS-L and NTS-S defined by the location of inserts from PAM. Specifically, the insert in NTS-L is located at 20–24nt from PAM, while the insert in NTS-S is located at 11–14nt from PAM. Red arrowheads indicate cleavage sites of LbCas12 proteins on target genomic DNA. Insert above the triangle means an exogenous restriction site is inserted as code for calculation of the rate of HDR. The length of ssDNA donors used in this study is less than 200nt, and the length of PCR amplicons is less than 250nt. The other two panels show the ratios of HDR:indel calculated from the averaged NGS data from NTS-L (middle panel) and NTS-S (bottom panel). The three beneficial mutants from the selection display much better activities in genome editing in HEK293 cells when donor NTS-L is used. Significance tests were carried out between each mutant and the wild-type protein. Values represent the replicate average ± standard error of the mean, where *n* = 2 for NTS-S experiments. For NTS-L experiments, *n* = 3 for EMX1-10856 and CCR5-457 and *n* = 4 for EMX1-4368 and VEGFA-1662. *P*-values were determined using two-sided Dunnett's test: * *P* < 0.05, ** *P* < 0.01, *** *P* < 0.001.

We purified these three evolved Cas12a proteins and analyzed their *in vitro* nuclease activities. Kinetic studies of *cis*-cleavage showed that the three new variants displayed improved cleavage efficiency, especially on the target strand (Figure 4A, B, Table 1, Supplementary Figure S4A, B). Of the three mutants, mut2B and mut2C had *cis*-cleavage activity comparable to the wildtype LbCas12a protein. Specifically, the initial rates of mut2B and mut2C are 6.22 min^−1^ and 6.96 min^−1^ on NTS, and 3.50 min^−^^1^ and 3.77 min^−1^ on TS, respectively. These values are comparable to those observed for wildtype LbCas12a (5.85 min^−1^ for NTS, 5.26 min^−1^ for TS). We also observed increased *trans*-ssDNase activity for the three mutants (Figure 4C, Supplementary Figure S4C), consistent with the inherent partial coupling of *cis* and *trans* activities by the RuvC domain. However, this *trans*-ssDNase activity of all three variants remained 10–300-fold slower relative to the wild-type LbCas12a (Figure 4C).

**Table 1.** Summary of initial rates of all of LbCas12a proteins

Protein	WT	mut2	mut2A	mut2B	mut2C	WT^a^	mut2C-W^a^	mut2C-WF^a^
cis-TS*/NTS	5.26	0.51	0.72	3.50	3.80	0.31	0.64	0.074
cis-TS/NTS*	5.85	3.19	4.38	6.22	6.96	0.16	0.27	0.27
trans-activity	6.30	N.D.	0.02	0.08	0.64	3.20	3.90	4.70

*: labeled strand; TS = target strand; NTS = non-target strand; WT = wild-type LbCas12a.

^a^ In *cis*-cleavage assays, 20 nM protein, 24 nM crRNA, and 40 nM target dsDNA were used. In *trans*-cleavage assays, 20 nM protein, 24 nM crRNA, 30 nM activator, and 120 nM target dsDNA were used.

Following biochemical analysis, we also investigated the genome editing activities of these engineered mutants, both for inducing small insertion or deletion mutations (indels) and for homology-directed repair (HDR). We wondered whether Cas12a enzymes with catalytic activity similar to wild-type protein for *cis*-dsDNA cleavage, but with minimal *trans*-ssDNase activity might favor genome editing by homology-directed repair (HDR). To test this possibility, we conducted genome-editing experiments using RNPs from the mutants of mut2A, mut2B, and mut2C by targeting different genes in HEK293T cells. We created suitable crRNAs for 19 different genomic targets. After 96 h of cell growth following RNP introduction into cells by nucleofection, genome editing efficiency was analyzed and quantified by next-generation sequencing (NGS). Overall, the evolved mutants significantly improved genome editing efficiency when compared to the starter mutant of mut2 (Supplementary Figure S4D1–5). In particular, mut2C was the best performing of the three evolved mutants and displayed similar levels of genome editing efficiency as measured by indels compared to the wild-type (Supplementary Figure S4D1). To measure the rates of HDR, exogenous restriction site (insert)-containing ssDNA oligonucleotides complementary to target or non-target strands were included as repair templates. Interestingly, we observed that these mutants significantly outperform wild-type Cas12a in HDR efficiency with donors (NTS-L) containing inserts at the PAM-distal region, which results in mismatches with crRNA at 20–24nt away from the PAM when Cas12a re-binds to the target site (Figure 4D, Supplementary Figure S4D4, D5).

### Reverse mutations in the bridge helix yield hyper-effective cas12a genome editors

**Figure 5.**
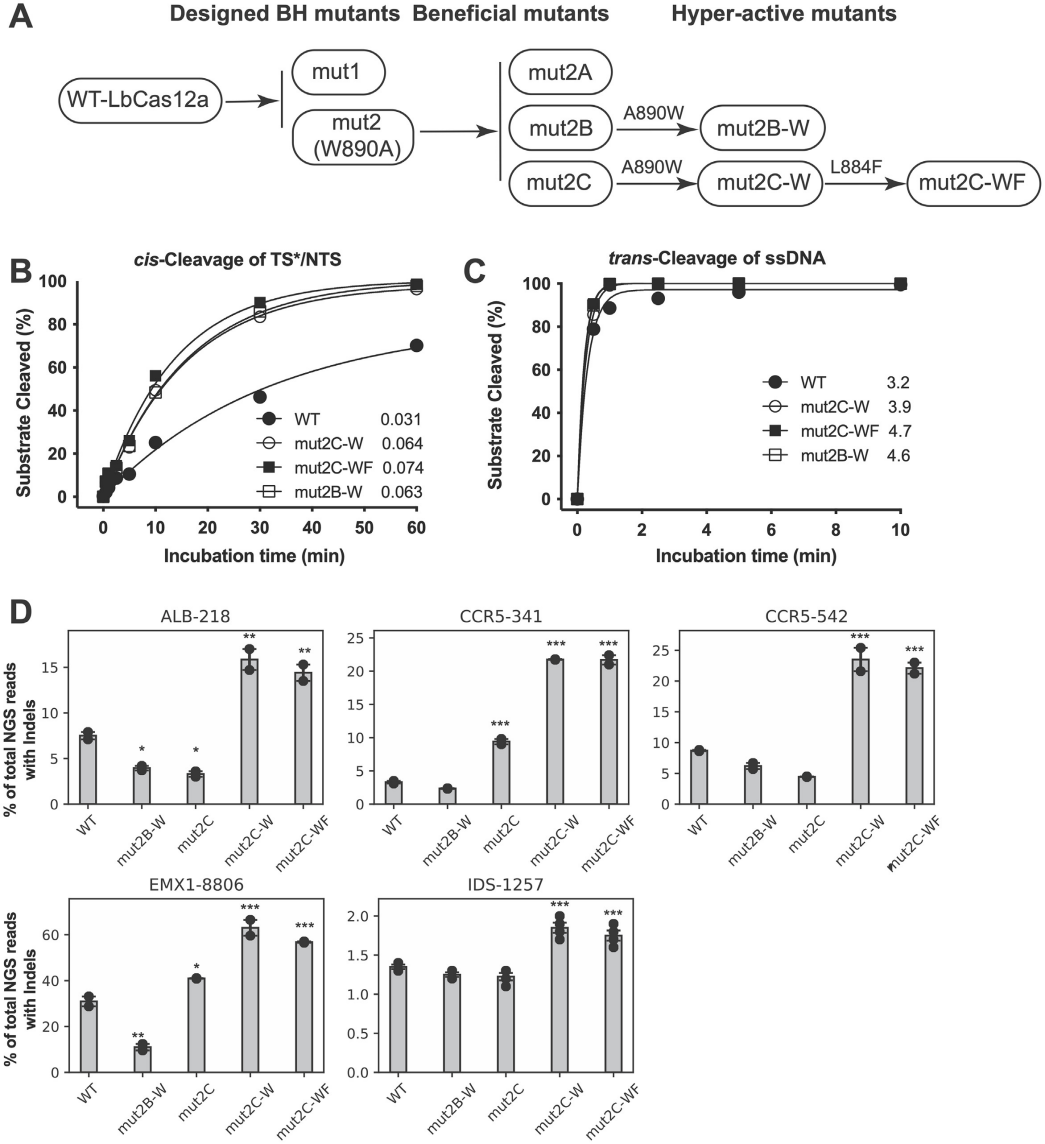
Hyper-effective LbCas12a proteins. (**A**) Schematic presentation of the overall pathway for generating beneficial and hyper-effective (HypE) LbCas12a mutants. These two HypE-mut2B-W and HypE-mut2C-W mutants are generated by restoring W890 in the corresponding beneficial mutants, respectively. HypE-mut2C-WF is a result of the restorations of both W890 and F884 in mut2C. (**B**, **C**) *In vitro* Kinetic studies of the *cis*- and *trans*-activities of the HypE-LbCas12a proteins using 20 nM protein, 24 nM crRNA, and 40 nM target-strand-labeled dsDNA. For *trans*-cleavage assays, 120 nM of labeled ssDNA was used. Each data point is averaged from two independent assays. In these kinetic studies, the labeled strand was fluorescently labeled at the 5’-end with FAM. Initial rates shown after each protein symbol. (**D**) Genome editing with HypE-mut2C-W and mut2C-WF. Selected loci are difficult to edit in HEK293 cells; significance tests were carried out between each mutant and wild-type LbCas12a. Values represent the replicate average ± standard error of the mean, where *n* = 2 for all experiments except for IDS-1257 (*n* = 4). *P*-values were determined using two-sided Dunnett's test: * *P* < 0.05, ** *P* < 0.01, *** *P* < 0.001.

The biochemical data from this study showed that mut2B and 2C are similarly active in DNA *cis*-cleavage compared to the wild-type protein, but with diminished *trans*-activity. Next, we wondered whether restoring the tryptophan residue (W890) in the bridge helix in these mutants might result in even more efficient Cas12a proteins. To test this hypothesis, we selected mut2B and mut2C to study the effect of W890 restoration. As mut2C has another mutation (F884L) in the bridge helix, we also introduced a double reversion, A890W and L884F, into this mutant. The three corresponding variants, mut2B-W, mut2C-W, and mut2C-WF, were cloned and purified for functional analysis (Figure 5A).

We found that the new variants with the restoration of W890 in bridge helix in the evolved mutants are more effective at targeted dsDNA cleavage, outperforming the wild-type protein by 2-fold when comparing initial reaction rates (Figure 5B, Table 1, Supplementary Figure S5A, B). Not surprisingly, their *trans*-cleavage activity against non-specific ssDNA was also increased when compared to the wild-type protein (Figure 5C, Supplementary Figure S5C, D). These results suggest that the mutations identified from directed evolution with the intact bridge helix improve both *cis* and *trans*-cleavage, and the tryptophan of W890 in the bridge helix plays a key role in the sequential activities displayed by Cas12a. Interestingly, the data from protein thermostability assays showed that both Apo proteins and RNPs of the evolved LbCas12a mutants are thermally labile (Supplementary Figure S6), indicating conformational differences from the wild-type protein. Furthermore, structure prediction by AlphaFold2 shows that the three active variants (mut2B-W, mut2C-W, and mut2C-WF) generated in this study predominantly adopt open conformations, whereas wild-type Cas12a predominantly adopts a closed conformation (Supplementary Figure S7). Again, the difference seen from this structure prediction between the wild-type and three active mutant proteins might represent structural changes in these mutants caused by the mutations.

We next tested whether the three improved Cas12a mutants (mut2B-W, mut2C-W, and mut2C-WF, Figure 5A) are capable of improved genome editing in mammalian cells. We selected five genomic loci, four of which have proven challenging for genome editing in prior experiments. Analysis of NGS data showed that mut2C-W and mut2C-WF significantly outperformed wild-type LbCas12a (*P* < 0.001). Specifically, they produced 2–5-fold improvements in genome editing efficiency relative to the wild-type LbCas12a (Figure 5D).

The results from genome editing together with the data from our DNA *in vitro* cleavage assays clearly showed that these Cas12a mutants of mut2C-W and mut2C-WF are improved Cas12a (iCas12a) editors that can be further developed as robust tools for both genome editing and diagnostics.

## DISCUSSION

Unlike Cas9, the Cas12a nucleases are not as widely utilized for genome editing due to lower efficiency and specificity (33,34). To overcome these obstacles, several studies using either structure-guided protein engineering (26,35) or directed evolution (25) have generated highly active variants of *Acidaminococcus Sp*. Cas12a (AsCas12a). To enrich the toolbox for genome editing, we used directed evolution to select highly active forms of LbCas12a from a starting mutant (mut2) for which *trans*-cleavage activity is diminished.

In this study, we found that the *cis*-cleavage activity of LbCas12a could be maintained while *trans*-activity was diminished by introducing mutations in the bridge helix, which connects the Rec and Nuc lobes of the protein. Key residues in the BH, which are highly conserved among different Cas12a orthologs, interact with the REC-II and RuvC domains and thus contribute to the closed-to-open conformational transitions of the LbCas12a RNP once the target DNA is bound (13,15,18). Our study showed that disrupting these interactions by introducing mutations at these sites, such as W890A, E880A, and R883A, significantly reduced the cleavage of the target strand (TS) and even abolished the indiscriminate *trans*-cutting function, with less impact on the cleavage of the non-target strand (NTS). The reduction of *cis-*cleavage activity on TS and abolishment of *trans*-cleavage activity could be from the reduction of trimming activity on NTS. It has been reported that abolishing or inhibiting NTS trimming activity in Cas12a proteins could eventually slow down the target-strand cleavage and *trans*-cutting events (13,14). Our results, together with the results from recent studies with FnCas12a (15,20), highlight the importance of the bridge helix in affecting functionally necessary conformational dynamics of the two major lobes. However, further biophysical investigations are required to identify the key conformational transitions affected by bridge helix perturbations for LbCas12a.

Genome-editing experiments from this study showed impaired editing efficiency induced by the W890A mutation in mut2. Reduced genome editing levels may result from altered conformational dynamics of the apo protein or binary complex, reduced product release by the Cas12a W890A mutant and/or reduced end-trimming activity on the NTS that could foster perfect DNA repair to maintain the unedited sequence. These hypotheses need to be tested with additional biochemical and biophysical experiments.

To improve the genome editing efficiency of mut2, we used directed evolution to obtain three variants, mut2A, mut2B, and mut2C, with improved *cis*-nuclease activity relative to mut2. The mutations in these evolved variants are located primarily in the Nuc lobe (distributed in RuvC, Nuc domains, and BH). The RuvC domain performs the key nuclease function, the BH region regulates protein conformational changes, and the Nuc domain is also known to be involved in precisely positioning the target strand of dsDNA prior to its cleavage event (9,11,18,20,36). Therefore, it is reasonable to speculate that the higher activities displayed in the selected variants might benefit from mutations in the Nuc lobe, which may help to restore the appropriate conformational transitions required for higher levels of *cis*-cleavage activity of Cas12a.

Genome editing data from this study showed that wild-type LbCas12a displays much lower HDR efficiency (with HDR donors containing exogenous mismatches with crRNA at 20–24nt away from PAM) when compared to W890A-containing mutants identified by directed evolution, although indel efficiency was similar between the groups. The lower HDR efficiency observed for wild-type LbCas12a could be due to re-binding and re-cleavage of the edited site, whereas such events might be reduced for W890A-containing mutants due to higher sensitivity to mismatches as observed for FnCas12a (15). This hypothesis is also supported by results from *in vitro* DNA cleavage assays, which show that the W890A-containing mutant proteins are more sensitive to mismatches at the region distal to the PAM compared to the wild-type protein (Supplementary Figure S8).

To further engineer improved Cas12a genome editors, we introduced the wild-type tryptophan residue at the end of the bridge helix into the evolved Cas12a variants since the intact BH and helix 1 of the RuvC II domain support functionally important conformational states import (13,37). Restoration of the intact bridge helix in the mutants selected from directed evolution resulted in three hyper-effective LbCas12a proteins of mut2B-W, mut2C-W, and mut2C-WF. These new LbCas12a variants displayed further improved activities, both in *in vitro* cleavage of target DNAs and in genome editing with HEK293T cells. Structural predictions using AlphaFold2 on wild-type Cas12a and mut2B-W, mut2C-W, and mut2C-WF suggest that wild-type Cas12a predominantly adopts a closed (catalytically inactive) conformation, whereas the three mutants predominantly adopt an open (catalytically active) conformation (Supplementary Figure 7). Furthermore, the active mutants showed reduced thermostabilities compared to wild-type Cas12a (Supplementary Figure 6), consistent with enhanced conformational dynamics and potentially enhanced enzymatic activity. In the future, it will be interesting to compare these Cas12a variants to other engineered enzymes, including AsCas12a Ultra, enAsCas12a, AsCas12a-Plus, and hyperCas12a (25,26,26,38,39).

Our results support the importance of the bridge helix in the regulation of Cas12a activities. Overall, the combined structural-guided engineering and evolutionary strategy described here has created improved Cas12a genome editors and may be applicable to other CRISPR-Cas enzymes in the future.

## DATA AVAILABILITY

The NGS dataset used in this study is available under accession number PRJNA898646 (https://www.ncbi.nlm.nih.gov/sra/PRJNA898646). The raw denaturing gel images are available from the corresponding author upon request. All other data needed to evaluate the conclusions in the paper are present in the main manuscript or Supporting Information. The information for raw indel and HDR values and the NGS filename are in Supplementary Table 2.

## Supplementary Material

gkac1192_Supplemental_Files

## Notes

Present address: Junjie Liu, Beijing Advanced Innovation Center for Structural Biology & Frontier Research Center for Biological Structure, School of Life Sciences, Tsinghua University, Beijing 100084, China.

Present address: Junjie Liu, Tsinghua-Peking Center for Life Sciences, School of Life Sciences, Tsinghua University, Beijing 100084, China.
