# Supplementary material for: Improved genome editing by an engineered CRISPR-Cas12a
Source: Nucleic Acids Res. 2022 Dec 20;50(22):12689–701. doi: 10.1093/nar/gkac1192 (PMC9825149; doi:10.1093/nar/gkac1192)
Supplement: gkac1192_Supplemental_Files [file gkac1192_supplemental_files.zip › Final Version_Supplementary_sFigures &Legends.pdf]

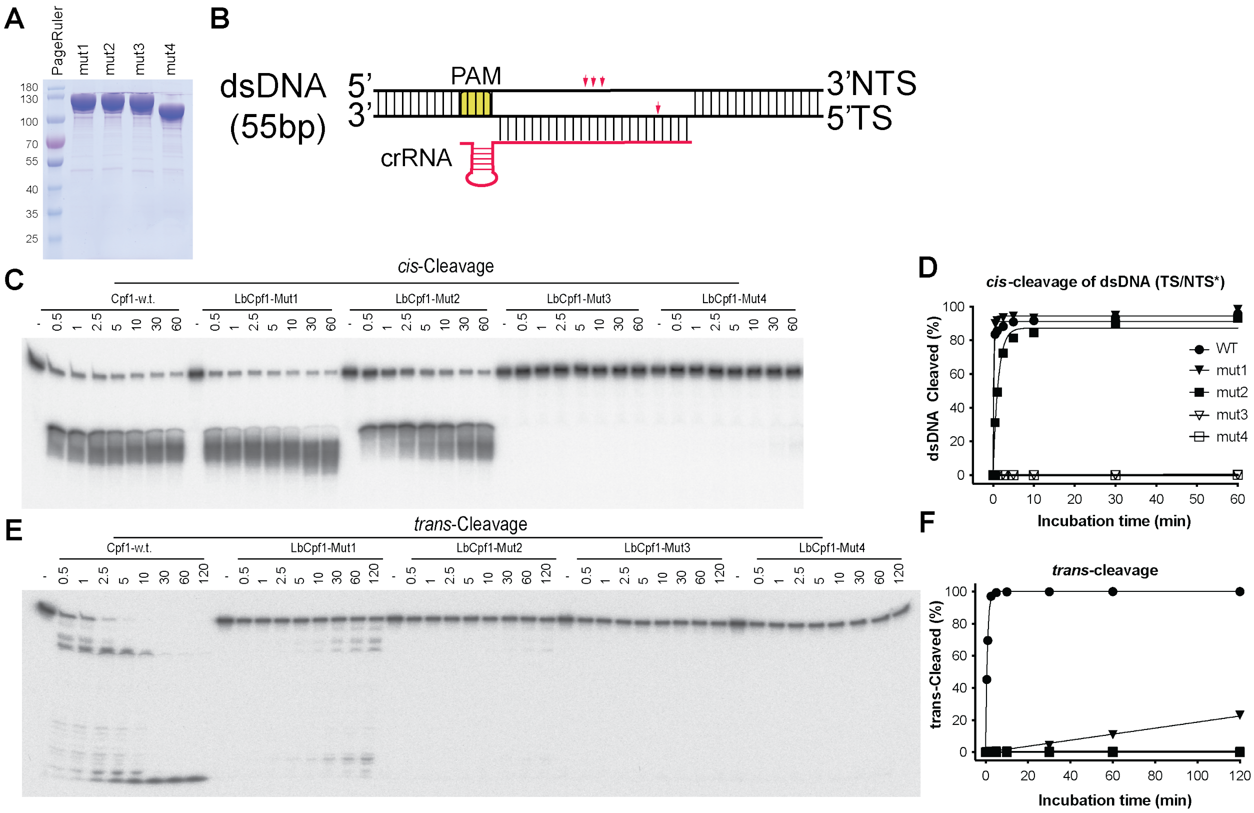

Supplementary Figure 1. Importance of the bridge helix (BH) of LbCas12a protein in regulating its nuclease activities. **A**. Purification of 4 LbCas12a mutant proteins. **B**. Schematic presentation of a target dsDNA (55bp) and 43nt CRISPR RNA (crRNA) containing a 19-base direct repeat region (loop domain) and a 24-base protospacer region complementary to the center of a 55-base pair (bp) dsDNA substrate in which the target region flanks the protospacer adjacent motif (PAM) of TTTA. **C**,**D**. *In vitro* kinetic studies of *cis*-cleavage activities by wild-type (WT) and four designed mutants, mut1-4. C shows an actual gel image and D is quantification from C. **E**,**F**. *In vitro* kinetic studies of *trans*- cleavage activities by wild-type (WT) and four designed mutants, mut1-4. E shows an actual gel image and F is quantification from E. In *cis*-cleavage assays, non-target strand of the dsDNA substrate was 5'-end-labeled with γ-^{32}P-ATP. In *trans*-cleavage assays, the ssDNA substrate was 5'-end-labeled with γ-^{32}P-ATP.

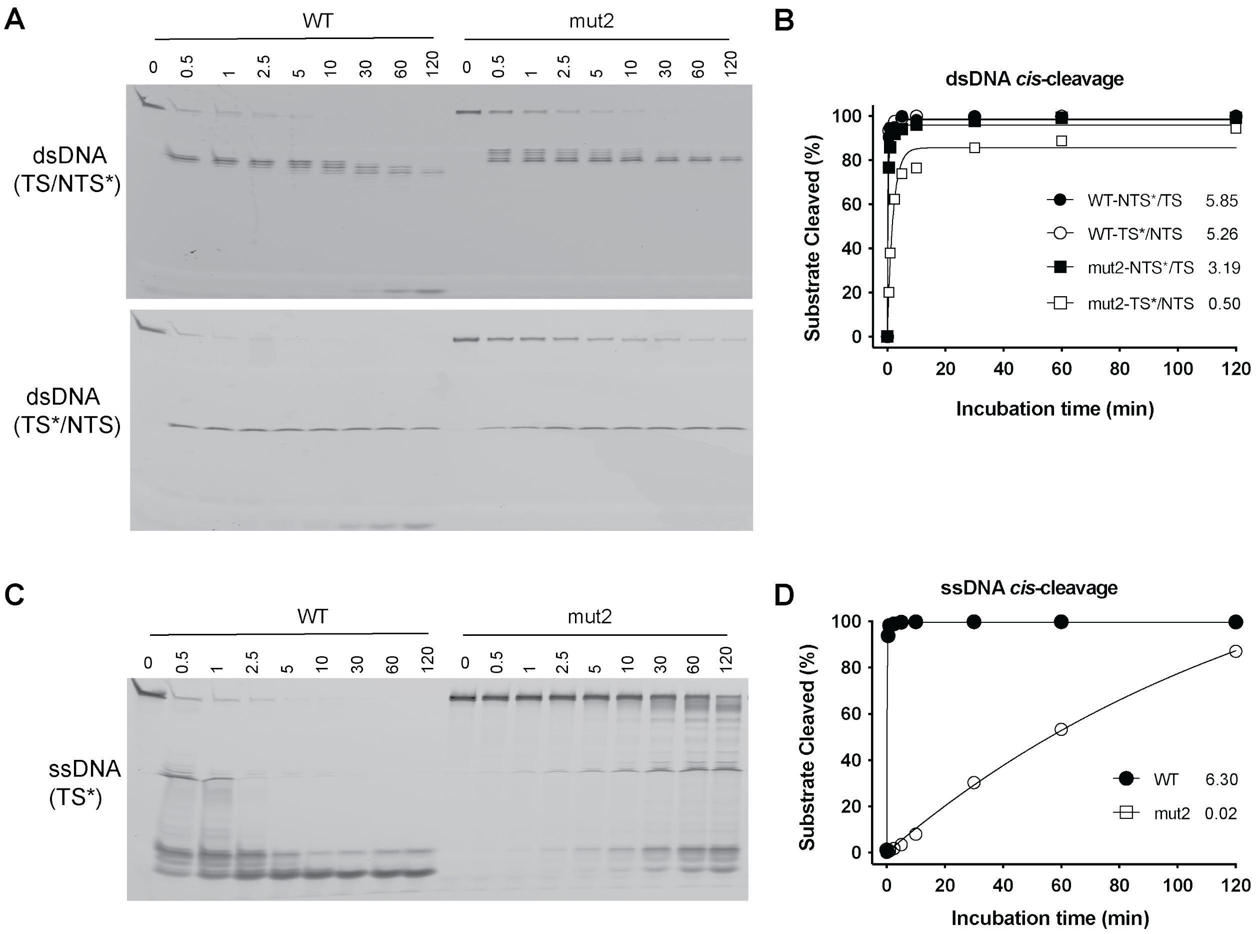

Supplementary Figure 2. Kinetic studies of mutational effect of W890A on the nuclease activities of LbCas12a proteins. **A**,**B**. *cis*-cleavage assays of dsDNA. A shows 2 actual images of the *cis*-cleavage activities on dsDNA. In TS/NTS* cleavage, trimming activity can be clearly seen with wild-type protein, but no with mut2 protein. B is quantifications from A. **C**,**D**. *cis*-cleavage assays of a ssDNA. C is an actual image of the *cis*-cleavage activities on a ssDNA and D is quantifications from D. TS=target strand; NTS= nontarget strand; *indicates labelled strand. mut2 is W890A point mutation of LbCas12a. In these kinetic studies, the labeled strand(*) was fluorescently labeled at 5-end with fluorophores of 5’6-FAM.

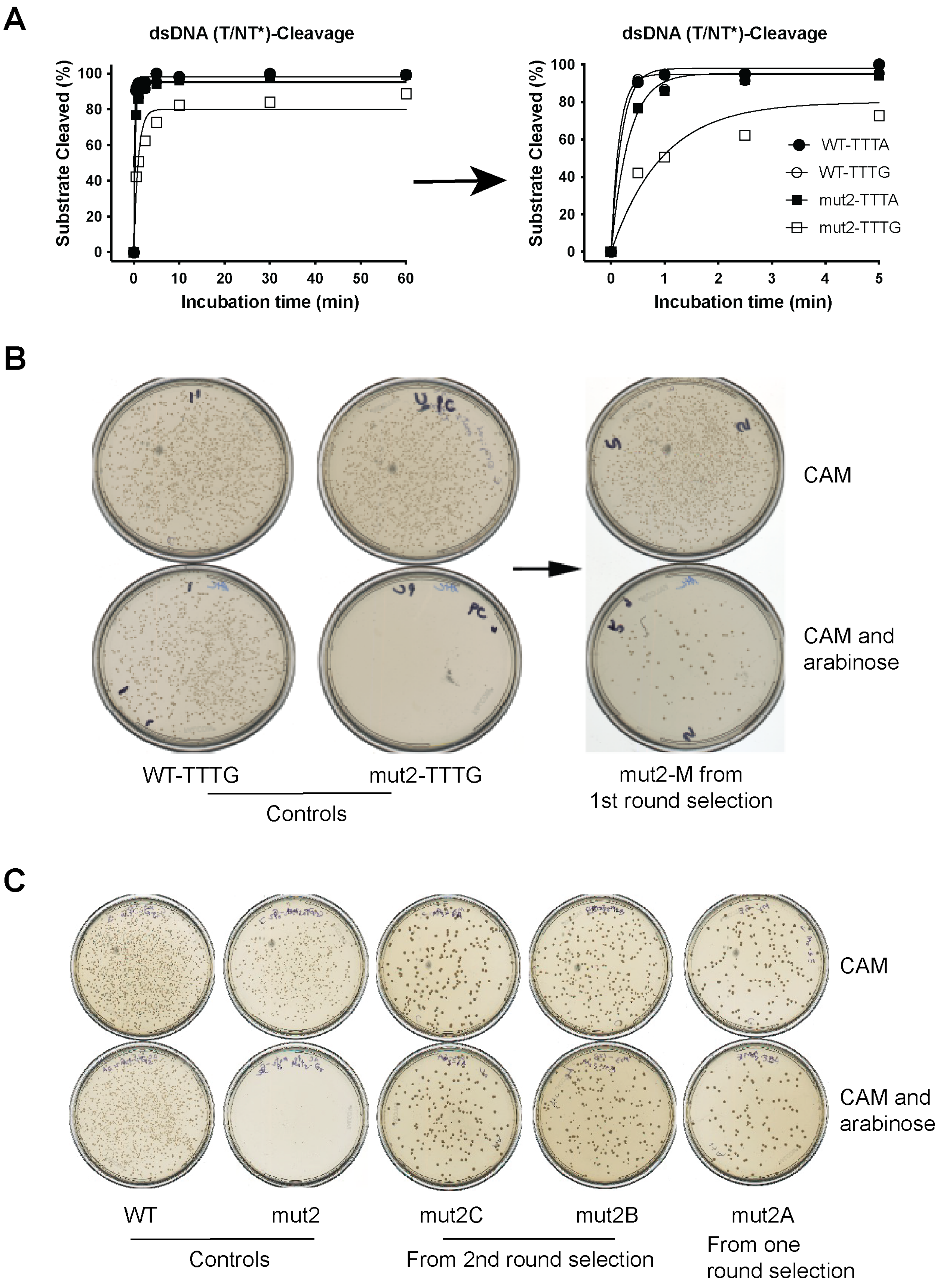
A
dsDNA (T/NT*)-Cleavage
Substrate Cleaved (%)
Incubation time (min)
dsDNA (T/NT*)-Cleavage
Substrate Cleaved (%)
Incubation time (min)
WT-TTTA
WT-TTTG
mut2-TTTA
mut2-TTTG
B
CAM
CAM and arabinose
WT-TTTG
mut2-TTTG
Controls
mut2-M from 1st round selection
C
CAM
CAM and arabinose
WT
mut2
Controls
mut2C
mut2B
From 2nd round selection
mut2A
From one round selection

Supplementary Figure 3. More active variants of mut2 selected by directed evolution. **A**. Effects of mut2 (W890A) on PAM usage. mut2 disfavors TTTG PAM which is used for the directed evolution selection. In these kinetic studies, the non-target strand was fluorescently labeled at 5-end with fluorophores of 5’6-FAM. **B**. First round selection of directed evolution. Two mutants were selected: mut2A contained mutations of E217A, E885V, W890A and I1028T and mut2M contains mutations of F863V, W890A, Q1108L, S1132T and S1214P. **C**. Second round selection using mut2M as a template. Two variants with higher activity from this round were selected: mut2B: K623R, F863V, W890A, Q1108L, S1132T, S1214P and mut2C: F863V, F884L, W890A, D952N, C965Y, V1011A, Q1108L, A1113V, S1132T, S1214P.

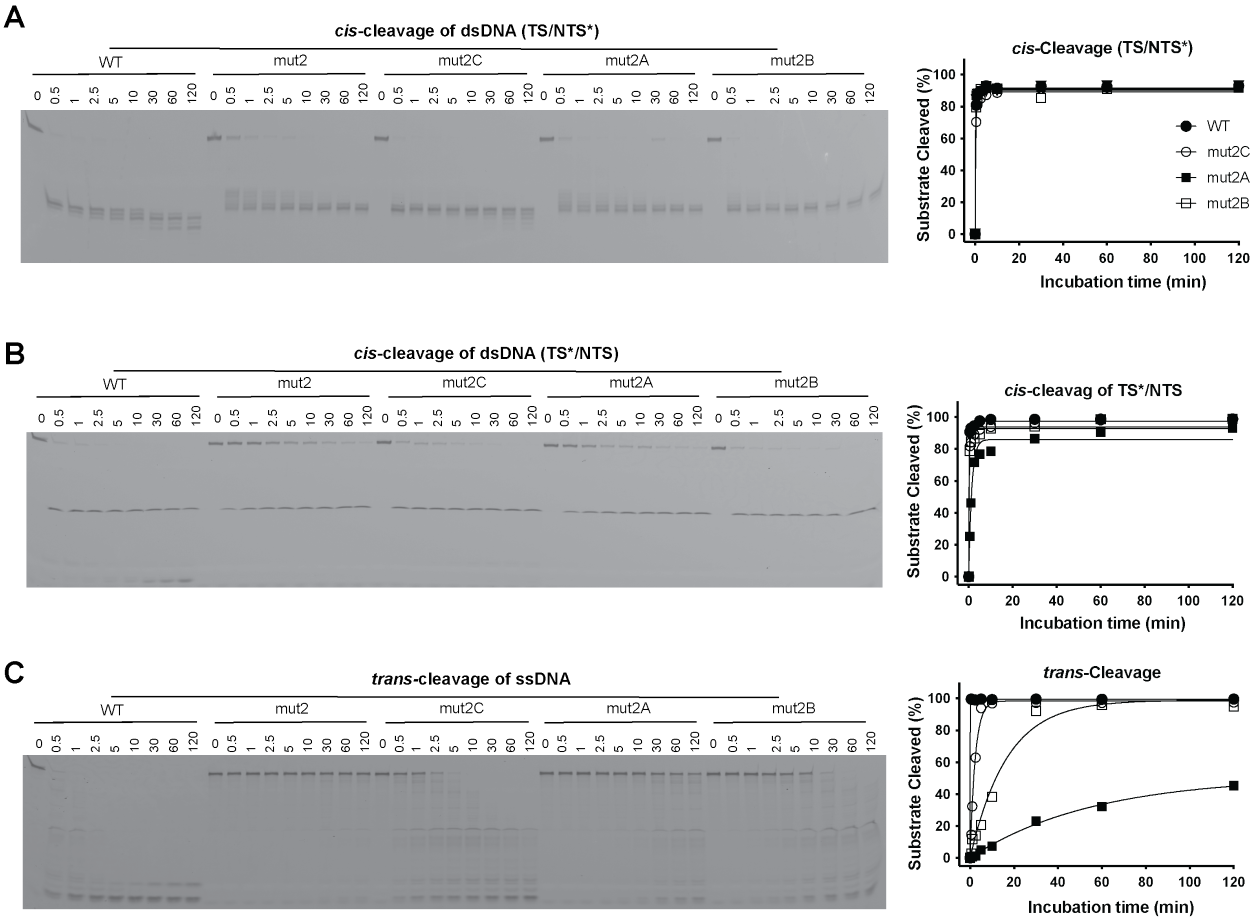

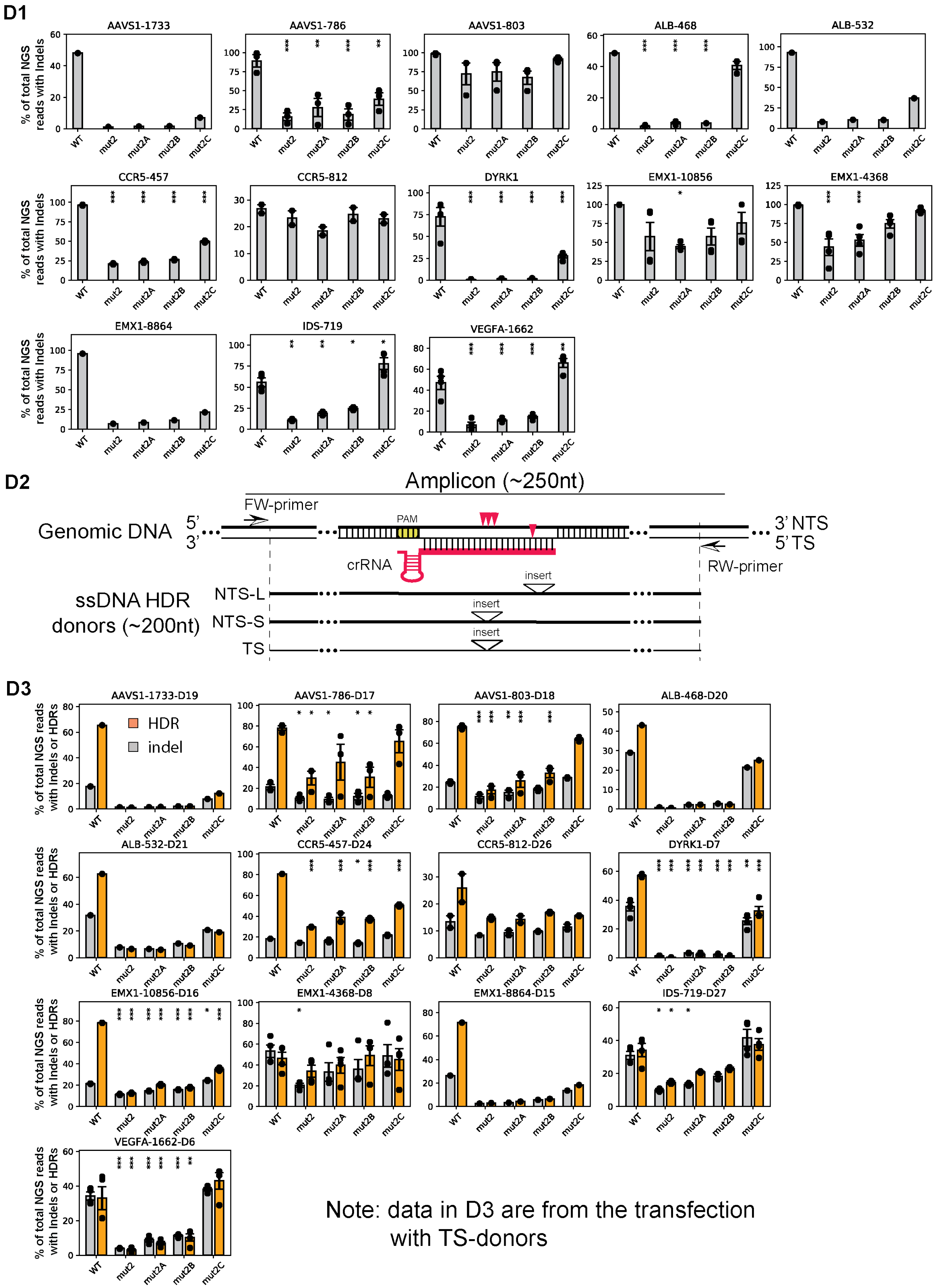

D1
AAVS1-1733
AAVS1-786
AAVS1-803
ALB-468
ALB-532
CCR5-457
CCR5-812
DYRK1
EMX1-10856
EMX1-4368
EMX1-8864
IDS-719
VEGFA-1662
% of total NGS reads with Indels
WT
mut2
mut2A
mut2B
mut2C
D2
Amplicon (~250nt)
FW-primer
Genomic DNA
5'
3'
PAM
3' NTS
5' TS
crRNA
RW-primer
insert
ssDNA HDR donors (~200nt)
NTS-L
NTS-S
TS
D3
AAVS1-1733-D19
AAVS1-786-D17
AAVS1-803-D18
ALB-468-D20
ALB-532-D21
CCR5-457-D24
CCR5-812-D26
DYRK1-D7
EMX1-10856-D16
EMX1-4368-D8
EMX1-8864-D15
IDS-719-D27
VEGFA-1662-D6
HDR
indel
% of total NGS reads with Indels or HDRs
Note: data in D3 are from the transfection with TS-donors

D4

Data in D4 are from the transfection with NTS-L donors

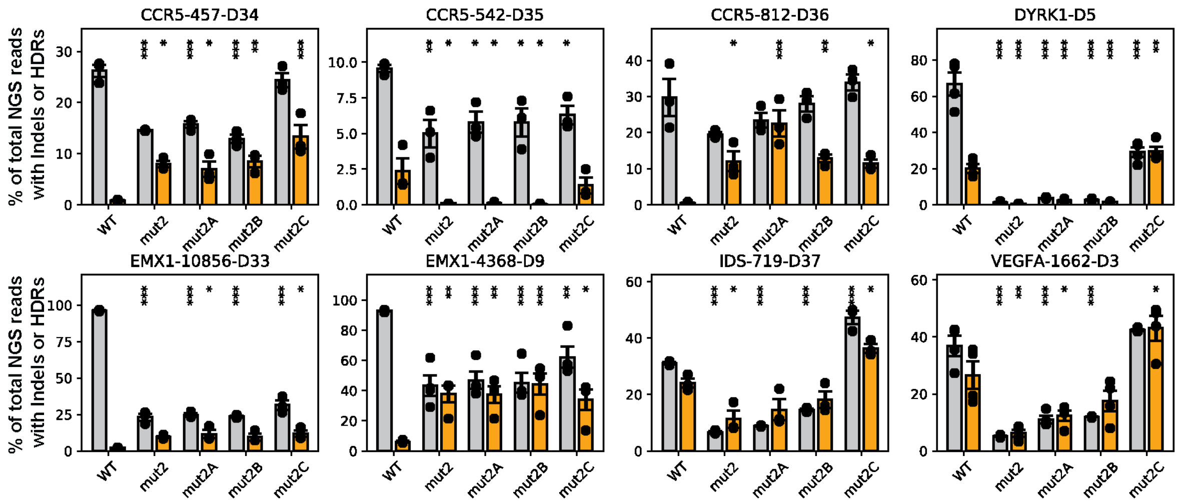

D5

Data in D5 are from the transfection with NTS-S donors

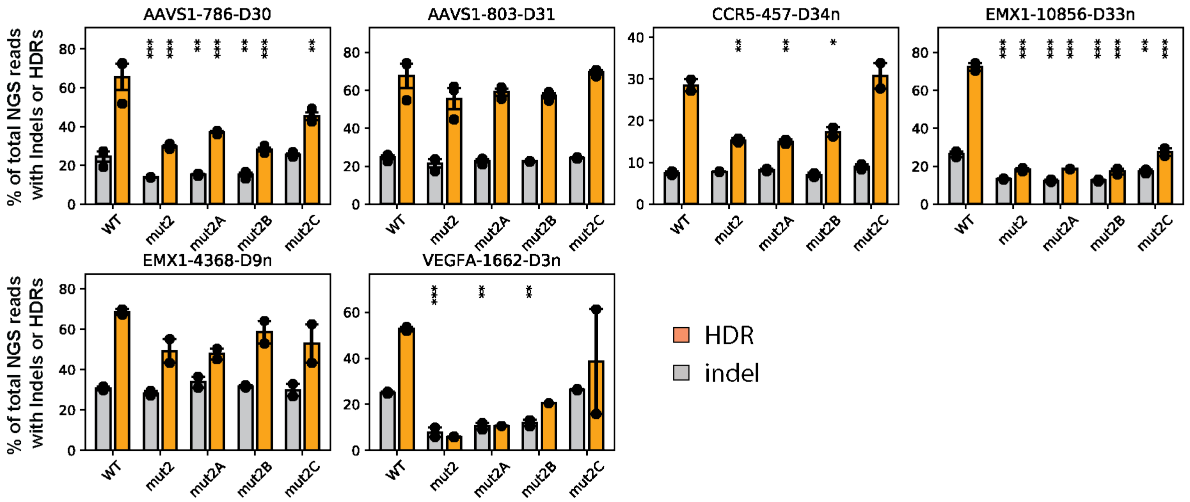

Supplementary Figure 4. Enhanced activity of mut2A-C *in vitro*. **A-C**. *In vitro* kinetic studies of the cleavage activities on NTS (**A**) and TS (**B**) in a dsDNA as well as the *trans*-cleavage activities (**C**). * indicates labeled strand. In these kinetic studies, the labeled strand(*) was fluorescently labeled at 5-end with fluorophores of 5'6-FAM. Left panels are actual cleavage images and right panels are their corresponding quantifications. All of 3 beneficial mutants from the directed evolution are more active than mut2. mut2B and mut2C display similar *cis*-activities on both strands as wild-type does, but their *trans*-activities are still much lower. **D**1-5. Genome editing in

HEK293T cells by the new variants selected from mut2 by directed evolution. Grey bars represent percentage (%) of total NGS reads with indels, while yellow bars represent percentage (%) of total NGS reads with HDRs. Each target significant test was carried out between each mutant and WT. Values represent the replicate average ± standard error of the mean, where n=2 for all experiments except for IDS-1257 (n=4). P-values were determined using two-sided Dunnett's test: * $P < 0.05$, ** $P < 0.01$, *** $P < 0.001$. **D**1 shows the rate (%) of Indels when the cells are transfected with only LbCas12a RNPs. Note that mut2C is the most active one among these mutants. **D**2 is schematic presentation showing the ssDNA donors for TS and NTS. For NTS donors, two kinds of NTS donors were designed: NTS-L and NTS-S defined by the location of inserts from PAM. Specifically, the insert in NTS-L is located at 20-24nt from PAM, while the insert in NTS-S is located at 11-14nt from PAM. Red arrowheads indicate cleavage sites of LbCas12 proteins on target genomic DNA. Insert above the triangle means an exogenous restriction site is inserted as code for calculation of the rate of HDR. The length of ssDNA donors used in this study is less than 200nt and the length of PCR amplicons is less 250nt; **D**3 shows the rates (%) of Indels and HDRs when the cells are transfected with both LbCas12a RNPs and TS ssDNA donors; **D**4 shows the rates (%) of Indels and HDRs when the cells are transfected with both LbCas12a RNPs and NTS-L ssDNA donors; **D**5 shows the rates (%) of Indels and HDRs when the cells are transfected with both LbCas12a RNPs and NTS-S ssDNA donors.

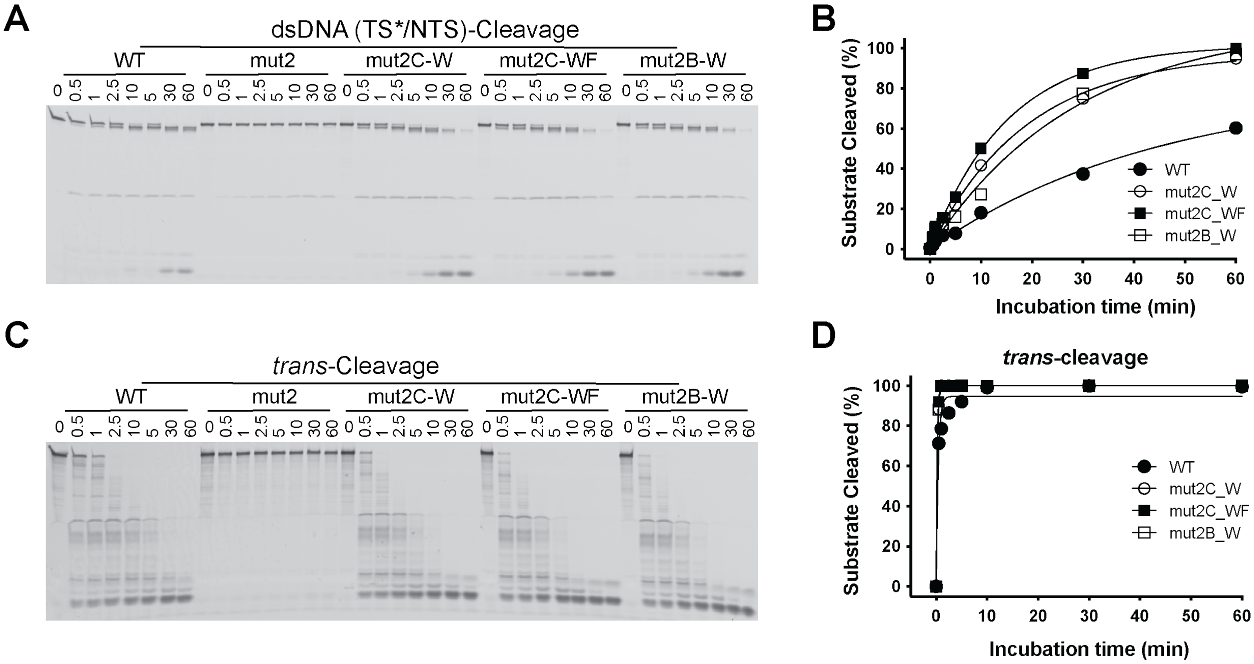

Supplementary Figure 5. Hyper-effective LbCas12a proteins. Restoration of tryptophan at W890 in the 2 beneficial mutants (mut2B and mut2C) generates hyper effective (HypE) mutants: mut2B-W, mut2C-W and mut2C-WF which contains two conversions F884 and W890. **A**,**B**. *In vitro* Kinetic studies of the *cis*-activities of the HypE-LbCas12a proteins. A is an actual cleavage image; B is quantification of A. The HypE-mutants are much more active in *cis*-dsDNA cleavage. Here, the *cis*-cleavage assays were carried out with high amount of target-strand-labeled dsDNA (TS*/NTS) since these mutants are very active. Specifically the reaction contains 20nM protein, 24nM crRNA, and 40nM target-strand-labeled dsDNA. **C**,**D**. *In vitro* kinetic studies of the *trans*-cleavage activities of the HypE-LbCas12a proteins. C is an actual cleavage image; D is quantification of C. Here, the *trans*-cleavage assays were carried out with 20nM protein, 24nM crRNA, 40nM activator dsDNA and 120nM of *trans*-ssDNA. In these kinetic studies, the labeled strand was fluorescently labeled at 5-end with fluorophores of 5'6-FAM.

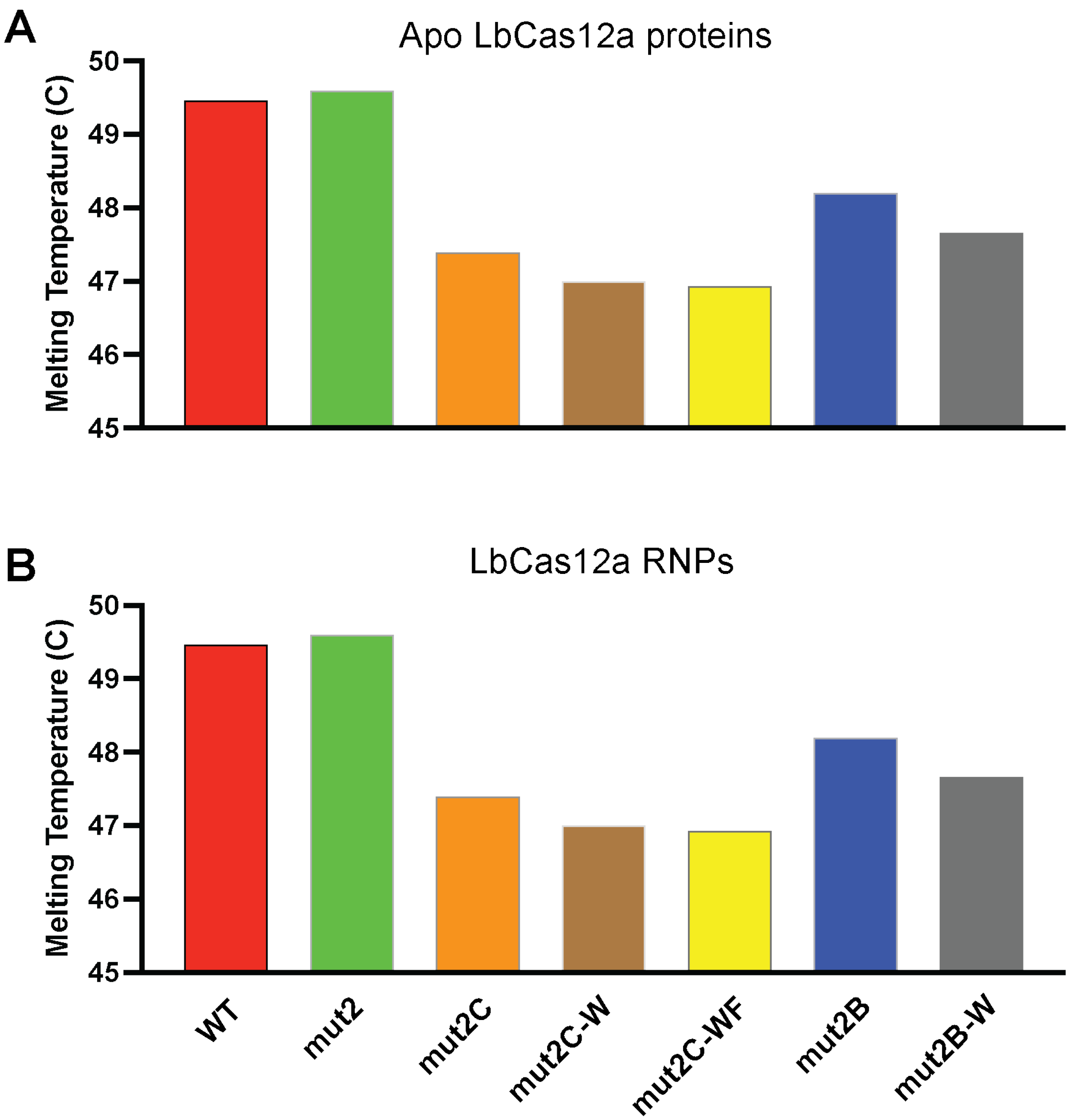

Supplementary Figure 6. Thermostability studies of LbCas12a mutant proteins and their RNPs. The reactions for analyzing the thermostability of LbCas12a proteins and their RNPs were performed based on the instruction from Protein Thermal Shift Dye kit (ThermoFisher Scientific). The protein melt fluorescent readings from CFX96 real-time System (BioRad) were directly recorded. Both Apo proteins and their RNPs of the evolved LbCas12a mutants are thermally labile.

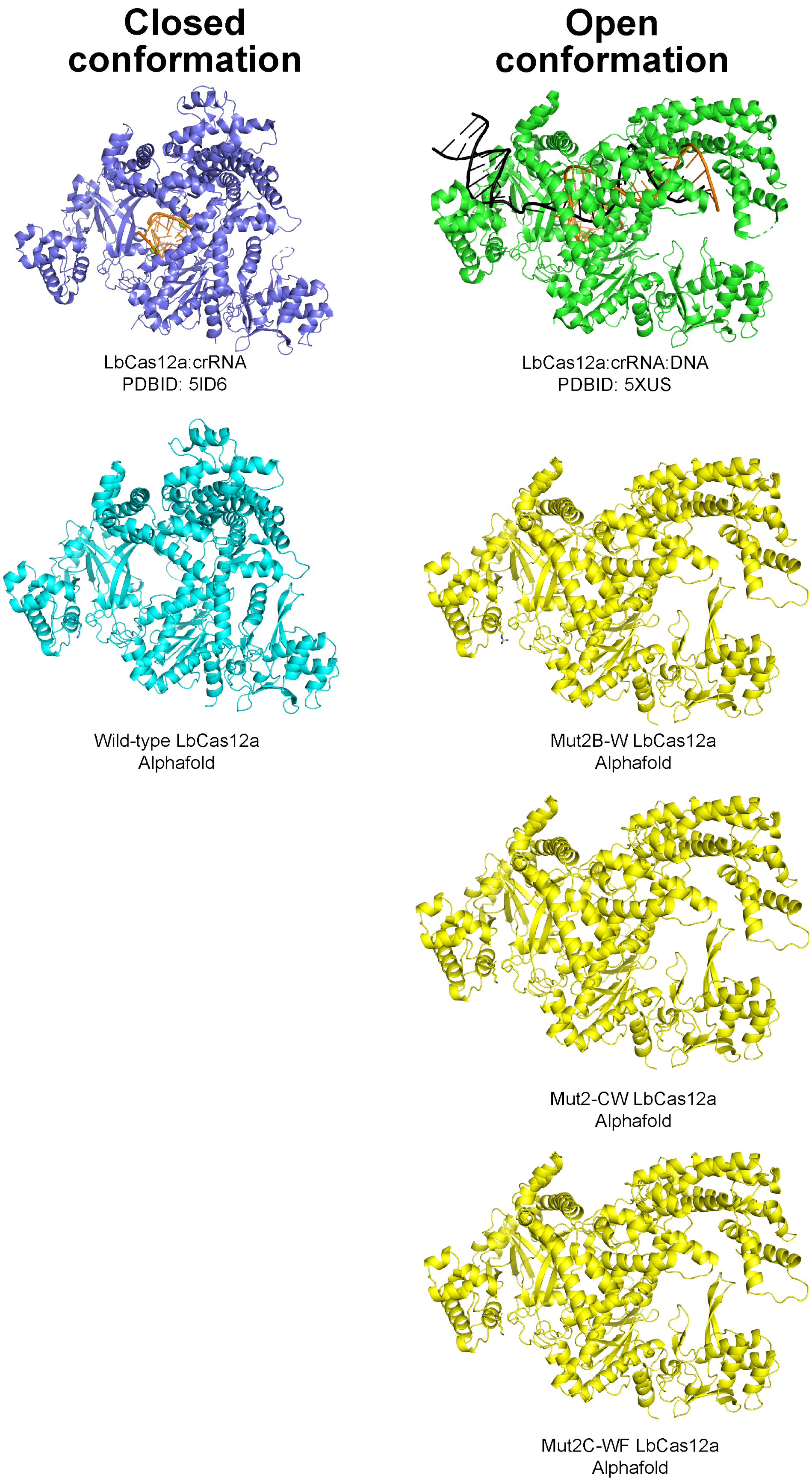
Closed conformation
Open conformation
LbCas12a:crRNA
PDBID: 5ID6
LbCas12a:crRNA:DNA
PDBID: 5XUS
Wild-type LbCas12a
Alphafold
Mut2B-W LbCas12a
Alphafold
Mut2-CW LbCas12a
Alphafold
Mut2C-WF LbCas12a
Alphafold

Supplementary Figure 7. Structural prediction of LbCas12a mutant proteins by AlphaFold2. The predicted structures show that the three active variants (mut2B-W, mut2C-W and mut2C-WF) generated in this study predominantly adopt open conformations whereas wild-type Cas12a predominantly adopts a closed conformation.

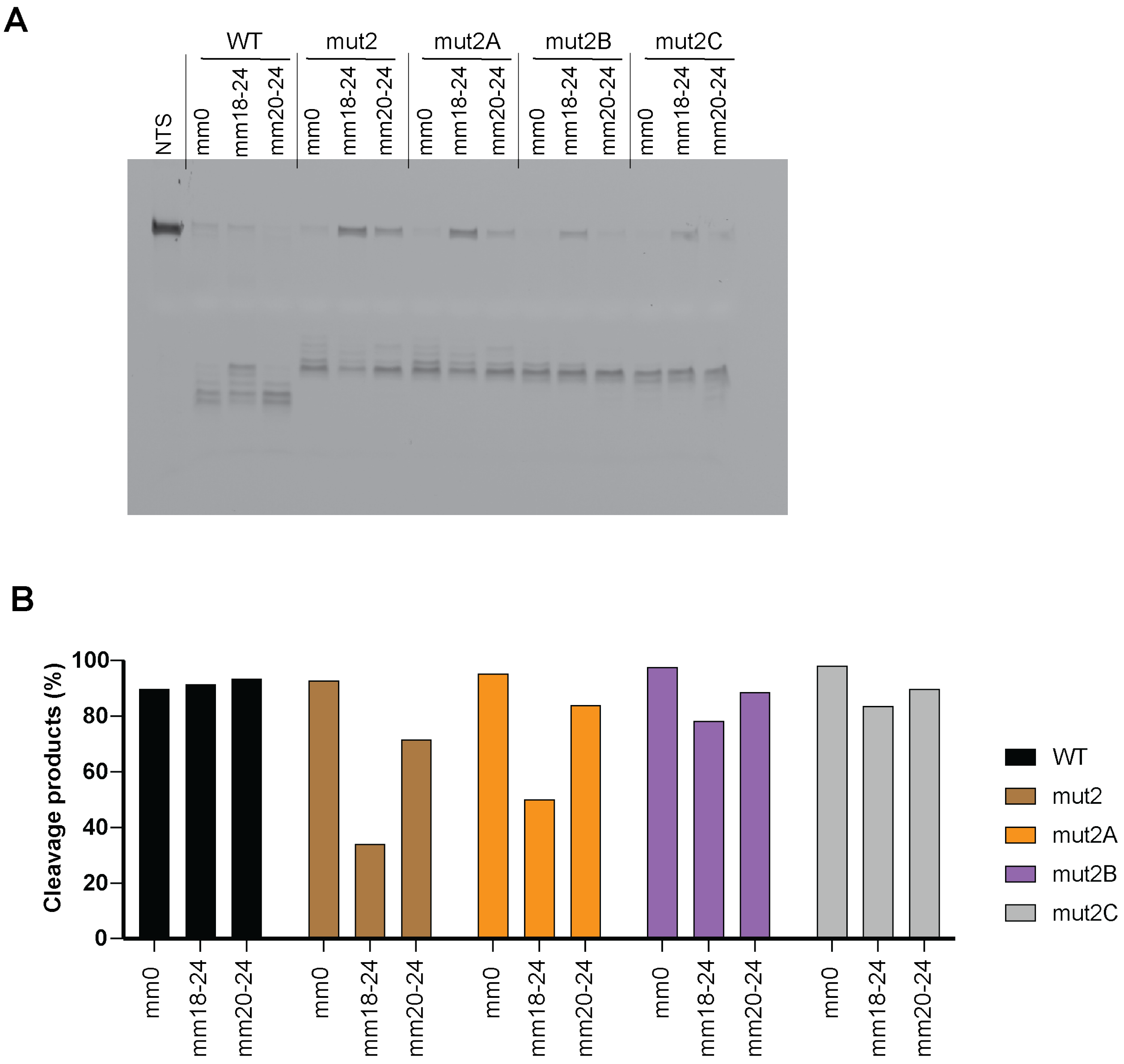

Supplementary Figure 8. *In vitro* DNA cleavage assays. In these cleavage assays, the NTS strand was fluorescently labeled at 5-end with fluorophores of 5'6-FAM. The W890A-containing

mutant proteins are more sensitive to mismatches at the region distal to the PAM compared to wild-type protein. Here, mm0=fully complementary dsDNA substrate; mm18-24=dsDNA substrate that carries mismatches at positions from 18 to 24 between crRNA and the DNA substrate; and mm20-24= substrate that carries mismatches at positions from 20 to 24 between crRNA and the DNA substrate. Cleavage assay were carried out with 60nM protein, 72nM crRNA and 10nM DNA substrate. The reaction was incubated at 37°C for 10 minutes.

Supplementary Table 1. Sequences of DNA and crRNA used in this study. The table contains DNA and crRNA Oligos used for in vitro studies and the sequences of DNA donors used in this study.

Supplementary Table 2. The information for raw indel and HDR values and the NGS filename. The table contains the rate of indel and HDR for each experiment with donor information and the corresponding original NGS file name.

The NGS dataset used in this study is available under accession number PRJNA898646 (https://www.ncbi.nlm.nih.gov/sra/PRJNA898646).
